# Cryo-EM structures and functional characterization of the murine lipid scramblase TMEM16F

**DOI:** 10.7554/eLife.44365

**Published:** 2019-02-20

**Authors:** Carolina Alvadia, Novandy K Lim, Vanessa Clerico Mosina, Gert T Oostergetel, Raimund Dutzler, Cristina Paulino

**Affiliations:** 1 Department of BiochemistryUniversity of ZurichZurichSwitzerland; 2 Department of Structural Biology at the Groningen Biomolecular Sciences and Biotechnology InstituteUniversity of GroningenGroningenNetherlands; National Institute of Neurological Disorders and Stroke, National Institutes of HealthUnited States; The University of Texas at AustinUnited States

**Keywords:** Cryo-EM, TMEM16F, lipid scramblase, ion conduction, electrophysiology, mechanism of action, Human, Mouse

## Abstract

The lipid scramblase TMEM16F initiates blood coagulation by catalyzing the exposure of phosphatidylserine in platelets. The protein is part of a family of membrane proteins, which encompasses calcium-activated channels for ions and lipids. Here, we reveal features of murine TMEM16F (mTMEM16F) that underlie its function as a lipid scramblase and an ion channel. The cryo-EM data of mTMEM16F in absence and presence of Ca^2+^ define the ligand-free closed conformation of the protein and the structure of a Ca^2+^-bound intermediate. Both conformations resemble their counterparts of the scrambling-incompetent anion channel mTMEM16A, yet with distinct differences in the region of ion and lipid permeation. In conjunction with functional data, we demonstrate the relationship between ion conduction and lipid scrambling. Although activated by a common mechanism, both functions appear to be mediated by alternate protein conformations that are at equilibrium in the ligand-bound state.

## Introduction

Lipid scramblases facilitate the movement of lipids between both leaflets of the bilayer (Bevers and Williamson, 2016; Nagata et al., 2016; Williamson, 2015), thereby dissipating the asymmetry present in the membrane. In contrast to ATP-dependent lipid flippases and floppases, scramblases are generally non-selective and do not require the input of energy (Pomorski and Menon, 2016). Lipid scrambling changes the properties of the bilayer and thus contributes to membrane synthesis, fusion and repair and the release of extracellular vesicles (Whitlock and Hartzell, 2017). The resulting exposure of negatively charged phospholipids to the outside is sensed by receptors that in turn initiate cellular responses such as blood coagulation and apoptosis (Nagata et al., 2016). In platelets, scrambling is mediated by the protein TMEM16F, which catalyzes the externalization of phosphatidylserine (PS) in response to an increase in the intracellular Ca^2+^ concentration to trigger blood clotting (Bevers and Williamson, 2016; Suzuki et al., 2013). Splicing mutations that lead to a truncated version of TMEM16F were found to cause Scott syndrome, a severe bleeding disorder in humans and dogs (Brooks et al., 2015; Castoldi et al., 2011; Lhermusier et al., 2011). TMEM16F belongs to the TMEM16 protein family, whose members either function as Ca^2+^ activated anion-selective channels (Caputo et al., 2008; Schroeder et al., 2008; Yang et al., 2008) or as Ca^2+^ activated lipid scramblases (Brunner et al., 2016; Falzone et al., 2018; Whitlock and Hartzell, 2017). The structure of the fungal TMEM16 homologue from *Nectria haematococca* (nhTMEM16), determined by X-ray crystallography, has defined the general architecture of the family and provided insight into the mechanism of lipid translocation (Brunner et al., 2014). In nhTMEM16, each subunit of the homodimeric protein contains a membrane-accessible polar furrow termed the ‘subunit cavity’, which provides a suitable pathway for the polar lipid headgroups on their way across the hydrophobic core of the bilayer (Bethel and Grabe, 2016; Brunner et al., 2014; Jiang et al., 2017; Lee et al., 2018; Stansfeld et al., 2015). This process closely resembles the ‘credit card mechanism’ for scrambling, which was previously postulated based on theoretical considerations (Pomorski and Menon, 2006). Conversely, single particle cryo-electron microscopy (cryo-EM) structures of murine TMEM16A (mTMEM16A), which instead of transporting lipids solely facilitates selective anion permeation (Dang et al., 2017; Paulino et al., 2017a; Paulino et al., 2017b), revealed the structural differences that underlie the distinct function of this branch of the TMEM16 family. In mTMEM16A, the rearrangement of an α-helix that lines one edge of the ‘subunit cavity’ in nhTMEM16 seals the membrane-accessible furrow, resulting in the formation of a protein-enclosed aqueous pore that is for a large part shielded from the bilayer. In both proteins, binding of Ca^2+^ mediates the activation of the permeation region contained in each subunit, which in mTMEM16A was shown to act as an independent entity (Jeng et al., 2016; Lim et al., 2016). Whereas the structures of nhTMEM16 and mTMEM16A have defined the architecture of two distantly related members of the family, TMEM16F appears, with respect to phylogenetic relationships, as intermediate between the two proteins. Although working as lipid scramblase (Suzuki et al., 2010; Watanabe et al., 2018), it is closer related to the ion channel mTMEM16A than to nhTMEM16 (Brunner et al., 2016; Falzone et al., 2018; Whitlock and Hartzell, 2017). Moreover, whereas scrambling-related ion conduction was found to be a feature of several family members (Lee et al., 2016; Malvezzi et al., 2018; Whitlock and Hartzell, 2016), TMEM16F is the only lipid scramblase for which rapidly activated calcium-dependent currents were recorded in excised patches (Yang et al., 2012). To better understand how the small sequence differences in TMEM16F give rise to its distinct functional properties, we determined the structure of murine TMEM16F (mTMEM16F) by cryo-EM in Ca^2+^-bound and Ca^2+^-free states, both in a detergent and in a lipid environment. In parallel, we characterized the lipid transport properties of mTMEM16F *in vitro* after reconstitution of the protein into liposomes, as well as ion conduction properties in transfected cells by electrophysiology. Collectively, our study reveals the architecture of mTMEM16F, defines conformational changes upon ligand binding and suggests potential mechanisms for ion and lipid conduction. In the most plausible scenario, both transport processes are activated by the same mechanism and mediated by distinct protein conformations, which are at equilibrium in a calcium-bound state.

## Results

### Functional properties of mTMEM16F

**Figure 1.**
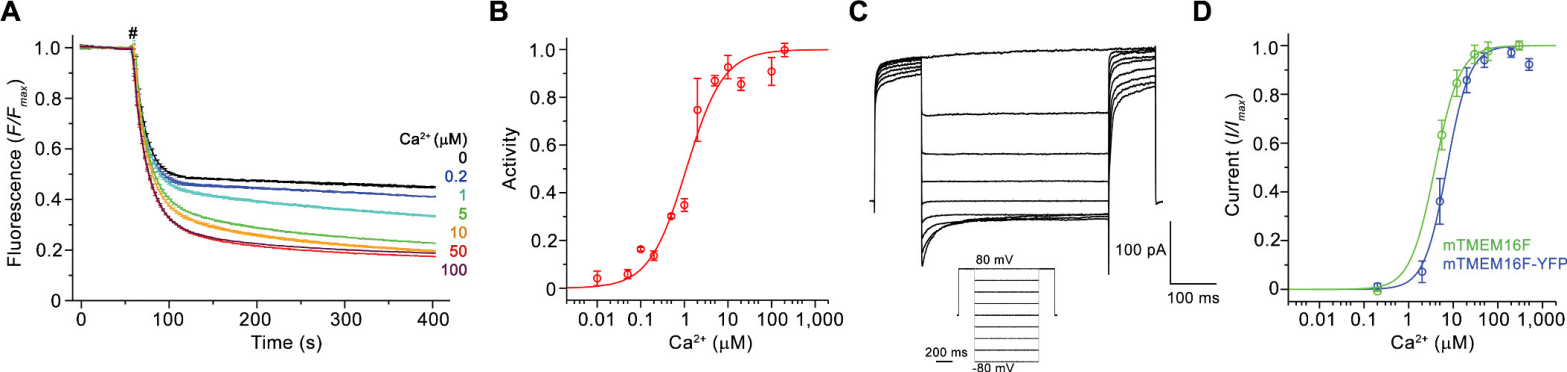
Functional characterization of mTMEM16F. (**A**) Ca^2+^-dependence of scrambling activity in mTMEM16F-containing proteoliposomes. Traces depict fluorescence decrease of tail-labeled NBD-PE lipids after addition of dithionite (#) at different Ca^2+^ concentrations. Data show averages of three technical replicates. (**B**) Ca^2+^ concentration-response relationship of mTMEM16F scrambling (EC_50_ = 1.1 μM, n = 1.0). Data show mean of six independent experiments from three protein reconstitutions. (**C**) Representative current traces at 200 μM Ca^2+^ recorded from inside-out patches excised from HEK293 cells transiently expressing a mTMEM16F-YFP fusion construct. Inset shows voltage protocol. (**D**) Ca^2+^ concentration-response relationship of currents recorded from inside-out patches of a stable HEK293 cell-line expressing mTMEM16F (green, EC_50_ = 3.9 μM, n = 1.6) and from HEK293 cells transiently expressing a mTMEM16F-YFP fusion construct (blue, EC_50_ = 7.2 μM, n = 1.7). Data show mean of five (stable cell-line) and seven biological replicates (transfected cells). B, D Solid lines show fit to a Hill equation. A, B, D, errors are s.e.m..

In our study, we explored the relationship between the structure of mTMEM16F, which shares a sequence identity of over 90% with its human orthologue, to its diverse functional properties. For this purpose, we have expressed mTMEM16F in HEK293 cells, purified it in the detergent digitonin in the absence of Ca^2+^ and, when indicated, added Ca^2+^ briefly before experiments (Figure 1—figure supplement 1A,B). To confirm that the purified protein has retained its function as a Ca^2+^-activated lipid scramblase, we investigated lipid transport with proteoliposomes using an assay that was previously established for fungal TMEM16 scramblases (Malvezzi et al., 2013; Ploier and Menon, 2016). This assay monitors the irreversible reduction of fluorescent lipids upon addition of the membrane-impermeable reagent dithionite to the outside of liposomes. The fluorescence rapidly decays to about half of its initial value in the absence of scrambling due to the bleaching of lipids already facing the outside, and it decreases further in a time-dependent manner in cases where a scramblase catalyzes lipid transport from the inner to the outer leaflet (Figure 1—figure supplement 1C). Our data reveal a Ca^2+^-induced activity, which, although dependent on the composition of proteoliposomes, facilitates transport of lipids with different headgroups, consistent with the broad lipid selectivity that was described for mTMEM16F and other TMEM16 scramblases (Brunner et al., 2014; Malvezzi et al., 2013; Suzuki et al., 2013) (Figure 1—figure supplement 1C–E). Activation of scrambling is fast, as evidenced by the immediate decay of the fluorescence upon addition of Ca^2+^ to the outside of mTMEM16F containing proteoliposomes (Figure 1—figure supplement 1F), and saturates with an EC_50_ for Ca^2+^ of about 1 µM (Figure 1A,B). Thus, our experiments demonstrate the function of purified and reconstituted mTMEM16F as a Ca^2+^-activated lipid scramblase.

**Figure 1—figure supplement 1.**
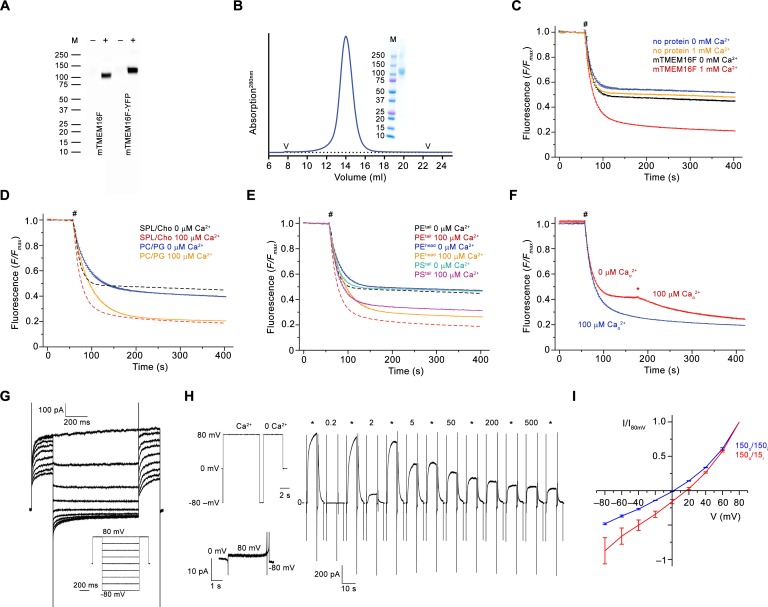
Biochemistry and functional characterization. (**A**) Western blot of stable cell-lines expressing mTMEM16F before (–) and after (+) induction with tetracycline. mTMEM16F and a C-terminal YFP fusion (mTMEM16F-YFP) were detected with a mouse monoclonal anti-Myc and a Peroxidase Affinipure goat anti-mouse IgG antibodies. (**B**) Size-exclusion chromatogram of purified mTMEM16F in digitonin. ‘V’ indicates void and dead volumes of the column. Inset shows SDS-Page of peak fractions. The molecular weight marker (M) and corresponding masses (kDa) are indicated. (**C**) Fluorescence-based lipid transport assay in liposomes containing soybean polar lipids and cholesterol used throughout the study. The fluorescence of tail-labeled NBD-PE was quenched by addition of dithionite (#) to the outside, which leads to a reduction of the fluorescence to about half in liposomes not containing any protein. The reduction in mTMEM16F-containing proteoliposomes in the absence of Ca^2+^ underlines the poor activity of the protein in absence of its ligand. The stronger decrease at high Ca^2+^ concentration reflects activity of mTMEM16F as lipid scramblase. The fluorescence at the plateau is due to lipids that are not accessible to dithionite. (**D**) Comparison of mTMEM16F scrambling activity in proteoliposomes of different lipid composition. Solid lines depict data from proteoliposomes containing a PC/PG mixture used for reconstitution into nanodiscs, which show a Ca^2+^-activated activity that proceeds with slower kinetics and larger basal activity compared to soybean and cholesterol containing liposomes (C, dashed line). (**E**) Scrambling of proteoliposomes with different labeled lipids. mTMEM16F is capable of scrambling tail-labeled NBD-PS (PS^tail^) and head-labeled NBD-PE (PE^head^), thus underlining its poor substrate specificity. Data for tail-labeled NBD-PE used for most experiments (c, dashed line) are shown for comparison. (**F**) Rapid activation of mTMEM16F is shown by addition of Ca^2+^ (*) 120 s after the addition of dithionite (#), which causes a further decrease of the fluorescence due to the exposure of labeled lipids that are transported from the inner leaflet following the activation of the scramblase (red). Proteoliposomes that were pre-incubated with Ca^2+^ on their outside before addition of dithionite are shown in blue for comparison. (C–F) Traces show average of three experiments from the same reconstitution. Errors are s.e.m.. # indicates addition of dithionite. (**G**) Representative current traces at 200 μM Ca^2+^ recorded from inside-out patches excised from HEK 293 cells stably expressing mTMEM16F. Inset shows voltage protocol. (**H**) Representative current response of mTMEM16F at different Ca^2+^ concentrations measured in excised inside-out patches. Numbers indicate Ca^2+^ concentration (µM), * indicates reference pulses recorded at 20 µM Ca^2+^ used for rundown correction. Individual sweeps are separated by a regular time interval (15–18 s) and are concatenated to form a continuous trace. Insets (left) shows voltage protocol used for each sweep in all concentration-response relationships throughout the study (top) and the leak currents at applied voltages in the absence of Ca^2+^ (bottom). (**I**) Normalized I-V relationships of mTMEM16F at symmetric (blue) and in a 10-fold gradient of the NaCl concentration (red) recorded from the instantaneous response following a voltage jump. The apparent decreased rectification in asymmetric conditions results from the low intracellular ion concentration. The osmolarity was adjusted by addition of NMDG-sulfate. The shift in the reversal potential in asymmetric conditions indicates a slight selectivity for cations over anions (P_Na+_/P_Cl-_=2.7 when assuming a negligible conductance of NMDG-sulfate). Data show averages of three biological replicates, errors are s.e.m.

Besides its capability to transport lipids, mTMEM16F was also reported to act as an ion channel (Kunzelmann et al., 2014; Scudieri et al., 2015; Yang et al., 2012; Yu et al., 2015). To recapitulate these properties, we have studied ion conduction in inside-out patches excised from HEK293 cells expressing mTMEM16F. As described previously, mTMEM16F mediates currents that are activated in response to elevated intracellular Ca^2+^ concentrations (Yang et al., 2012) (Figure 1C,D; Figure 1—figure supplement 1G). Analogous to the Ca^2+^-dependence of scrambling, the activation of ion conduction is rapid and currents (recorded at 80 mV) saturate with an EC_50_ of 4–7 µM. Similar results were obtained from the stable cell-line expressing mTMEM16F, used for reconstitution and structure determination, and from cells that were transfected with a construct containing a C-terminal fusion to YFP, which was used for the further characterization of mTMEM16F by electrophysiology (Figure 1D; Figure 1—figure supplement 1H). The Hill coefficient of about 1.7 for both mTMEM16F constructs emphasizes the cooperativity of the process. Unlike for the anion-selective mTMEM16A, the currents are slightly selective for cations over anions and they retain their strong outward rectification in the entire ligand-concentration range (Figure 1C; Figure 1—figure supplement 1G,I). Whereas the mild rectification of instantaneous currents is likely a consequence of conduction through an asymmetric pore (Paulino et al., 2017b), the presence of time-dependent relaxations in response to changes in the transmembrane voltage and the increased rectification at steady-state reflect a voltage-dependent transition that is not obviously linked to ligand binding (Figure 1C; Figure 1—figure supplement 1G). As the activation of ion conduction and lipid scrambling occurs in a very similar ligand concentration range, both processes likely rely on the same mechanism of Ca^2+^-activation.

### Structural characterization

**Figure 2.**
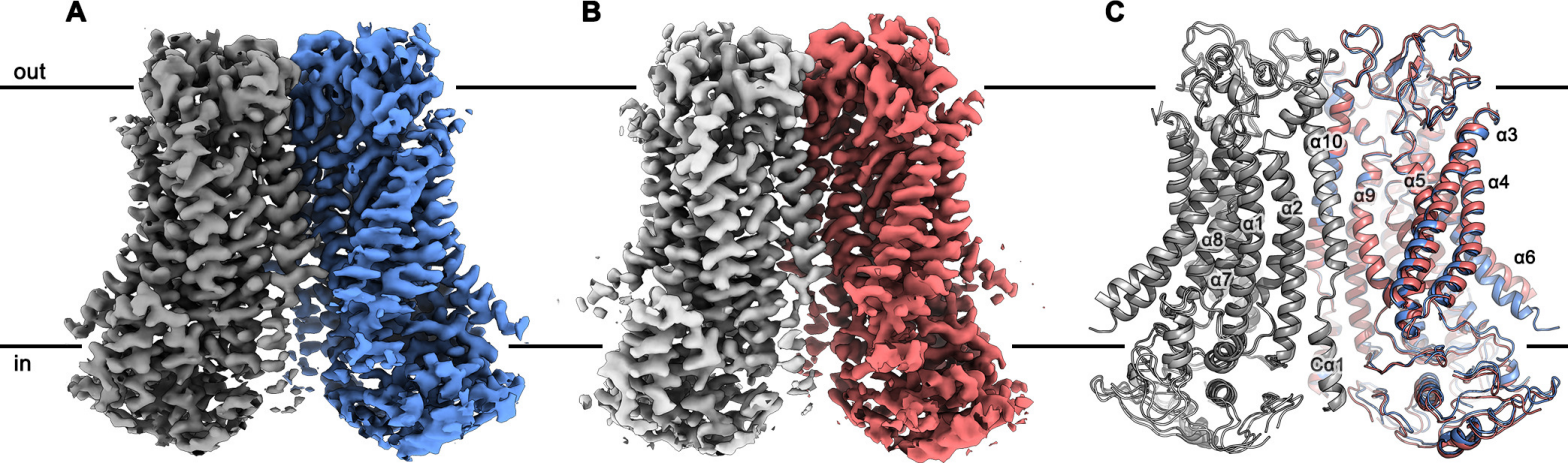
mTMEM16F structures. Cryo-EM map of mTMEM16F in digitonin in presence (**A**) and absence (**B**) of calcium at 3.2 Å and 3.6 Å, respectively. (**C**) Cartoon representation of a superposition of the Ca^2+^-bound (blue and grey) and Ca^2+^-free mTMEM16F (magenta and light grey) dimer. Both structures superimpose with a root mean square deviation (RMSD) of 1.2 Å. Transmembrane helices are labelled and the membrane boundary is indicated.

Since mTMEM16F combines the functional properties of a lipid scramblase and an ion channel, we were interested in the architectural features that distinguishes it from the anion-selective channel mTMEM16A and the fungal scramblase nhTMEM16. For that purpose, we determined structures of mTMEM16F in the absence and presence of Ca^2+^ (Table 1). Structures of the protein in the detergent digitonin were determined for Ca^2+^-bound and Ca^2+^-free states by cryo-EM at 3.2 Å and 3.6 Å, respectively (Figure 2; Figure 2—figure supplements 1 and 2; Videos 1 and 2). Both structures are very similar, except for conformational differences at the Ca^2+^-binding site and the ‘subunit cavity’ (Figure 2C).

**Figure 2—figure supplement 1.**
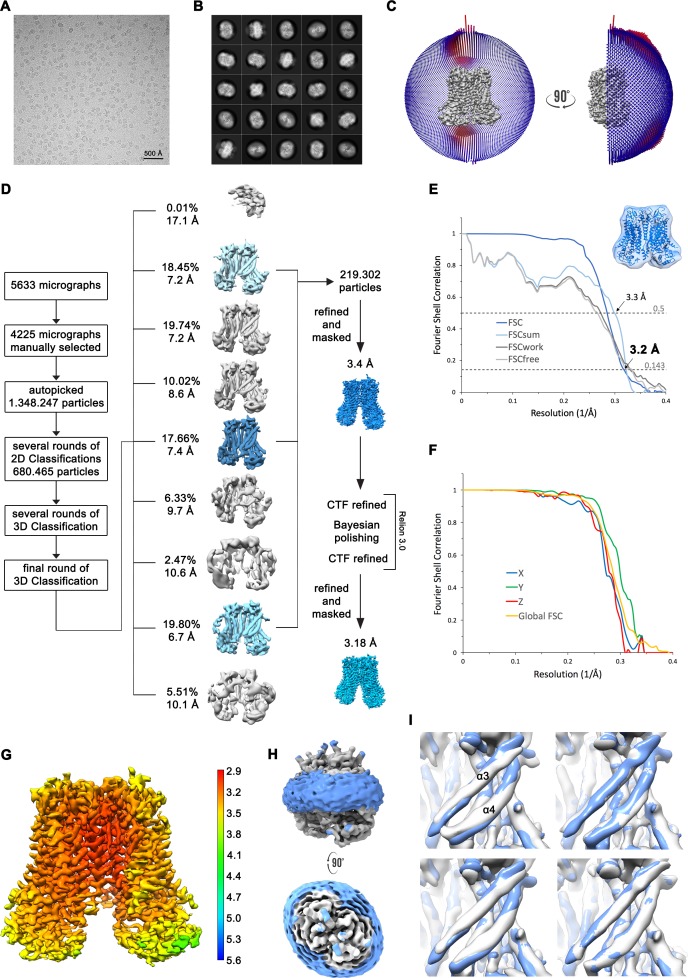
Structure determination of mTMEM16F in complex with Ca^2+^ in digitonin. (**A**) Representative cryo-EM image and (**B**) 2D-class averages of vitrified mTMEM16F in a Ca^2+^-bound state in digitonin. (**C**) Angular distribution plot of particles included in the final C2-symmetric 3D reconstruction. The number of particles with their respective orientation is represented by length and color of the cylinders. (**D**) Image processing workflow. (**E**) FSC plot used for resolution estimation and model validation. The gold-standard FSC plot between two separately refined half-maps is shown in dark blue and indicates a final resolution of 3.18 Å. FSC validation curves for FSC_sum_, FSC_work_ and FSC_free_, as described in Materials and methods, are shown in light blue, dark grey and light grey, respectively. A thumbnail of the mask used for FSC calculation overlaid on the atomic model is shown in the upper right corner and thresholds used for FSC_sum_ of 0.5 and for FSC of 0.143 are shown as dashed lines. (**F**) Anisotropy estimation plot of the final map. The global FSC curve is represented in yellow. The directional FSCs along the x, y and z axis displayed in blue, green and red, respectively, are indicative for an isotropic dataset. (**G**) Final reconstruction map colored by local resolution as estimated by Relion. (**H**) Representation of the map including the detergent micelle (low-pass filtered to 6.5 Å) reveals no noticeable distortion of the micelle (blue). (**I**) Analysis of structural heterogeneity, by subjecting the final dataset to another round of 3D classification, reveals alternative conformations of α3 and α4. Shown are superpositions of obtained 3D classes (grey) on the final cryo-EM map (blue), low-pass filtered to 6.5 Å.

**Figure 2—figure supplement 2.**
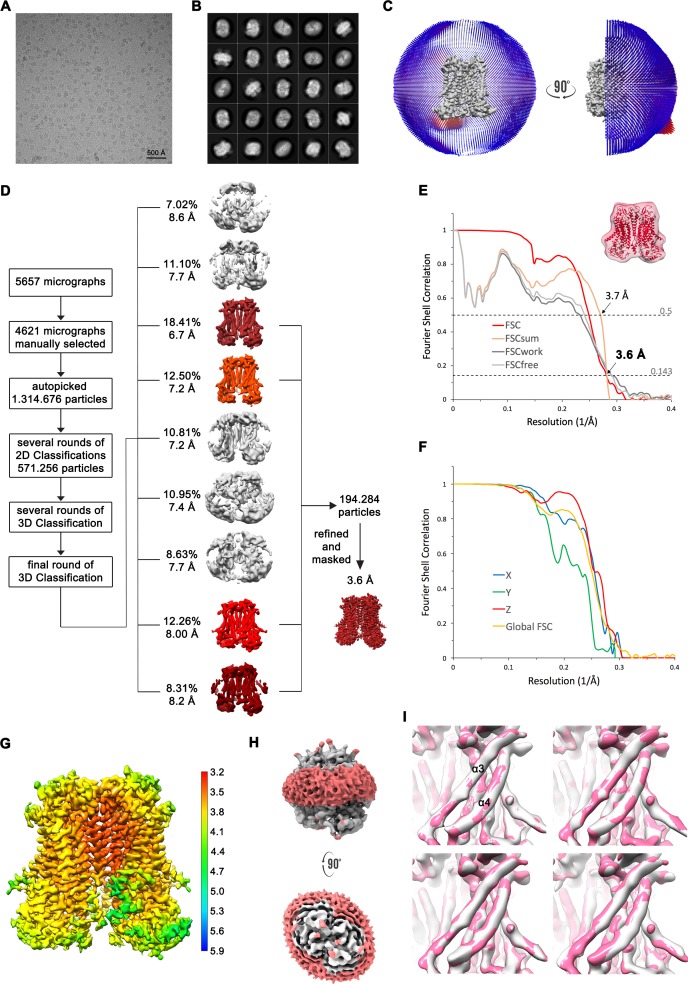
Structure determination of mTMEM16F in absence of Ca^2+^ in digitonin. (**A**) Representative cryo-EM image and (**B**) 2D-class averages of vitrified mTMEM16F in a Ca^2+^-free state in digitonin. (**C**) Angular distribution plot of particles included in the final C2-symmetric 3D reconstruction. The number of particles with their respective orientation is represented by length and color of the cylinders. (**D**) Image processing workflow. (**E**) FSC plot used for resolution estimation and model validation. The gold-standard FSC plot between two separately refined half-maps is shown in red and indicates a final resolution of 3.6 Å. FSC validation curves for FSC_sum_, FSC_work_ and FSC_free_, as described in Materials and methods, are shown in orange, dark grey and light grey, respectively. A thumbnail of the mask used for FSC calculation overlaid on the atomic model is shown in the upper right corner and threshold used for FSC_sum_ of 0.5 and for FSC of 0.143 are shown as dashed lines. (**F**) Anisotropy estimation plot of the final map. The global FSC curve is represented in yellow. The directional FSCs along the x, y and z axis shown in blue, green and red, respectively, are indicative for a largely isotropic dataset. (**G**) Final reconstruction map colored by local resolution as estimated by Relion. (**H**) Representation of the map including the detergent micelle (low-pass filtered to 6.5 Å) reveals no noticeable distortion of the micelle (red). (**I**) Analysis of structural heterogeneity, by subjecting the final dataset to another round of 3D classification, reveals no alternative conformations of α3 and α4. Shown are superpositions of obtained 3D classes (grey) on the final cryo-EM map (magenta), low-pass filtered to 6.5 Å.

**Figure 2—figure supplement 3.**
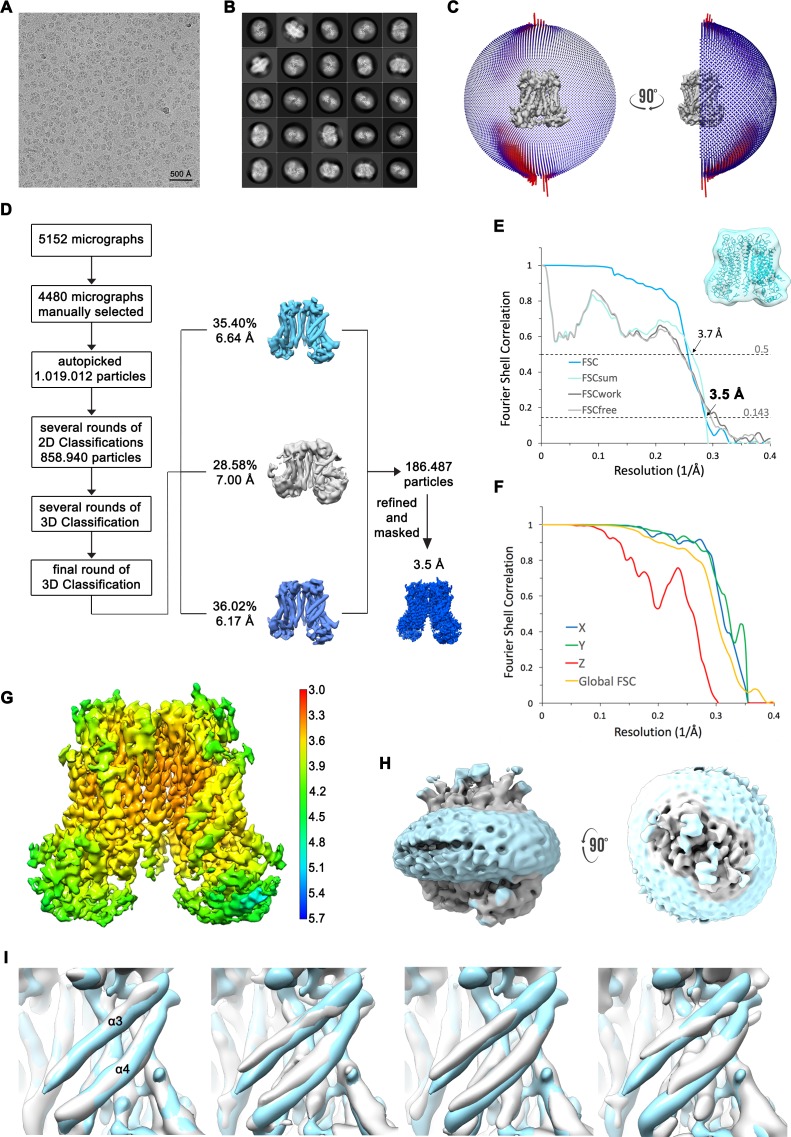
Structure determination of mTMEM16F in complex with Ca^2+^ in nanodisc. (**A**) Representative cryo-EM image and (**B**) 2D-class averages of vitrified mTMEM16F in a Ca^2+^-bound state in nanodiscs. (**C**) Angular distribution plot of particles included in the final C2-symmetric 3D reconstruction. The number of particles with their respective orientation is represented by length and color of the cylinders. (**D**) Image processing workflow. (**E**) FSC plot used for resolution estimation and model validation. The gold-standard FSC plot between two separately refined half-maps is shown in blue and indicates a final resolution of 3.5 Å. FSC validation curves for FSC_sum_, FSC_work_ and FSC_free_, as described in Materials and methods, are shown as light blue, dark grey and light grey, respectively. A thumbnail of the mask used for FSC calculation overlaid on the atomic model is shown in the upper right corner and threshold used for FSC_sum_ of 0.5 and for FSC of 0.143 are shown as dashed lines. (**F**) Anisotropy estimation plot of the final map. The global FSC curve is represented in yellow. The directional FSCs along the x, y and z axis are shown in blue, green and red, respectively, and indicate a dataset that is to some extent anisotropic. (**G**) Final reconstruction map colored by local resolution as estimated by Relion. (**H**) Representation of the map including the nanodisc (low-pass filtered to 6.5 Å) reveals no noticeable distortion of the nanodisc (light blue). (**I**) Analysis of structural heterogeneity, by subjecting the final dataset to another round of 3D classification, reveals alternative conformations of α3 and α4. Shown are superpositions of obtained 3D classes (grey) on the final cryo-EM map (light blue), low-pass filtered to 6.5 Å.

**Figure 2—figure supplement 4.**
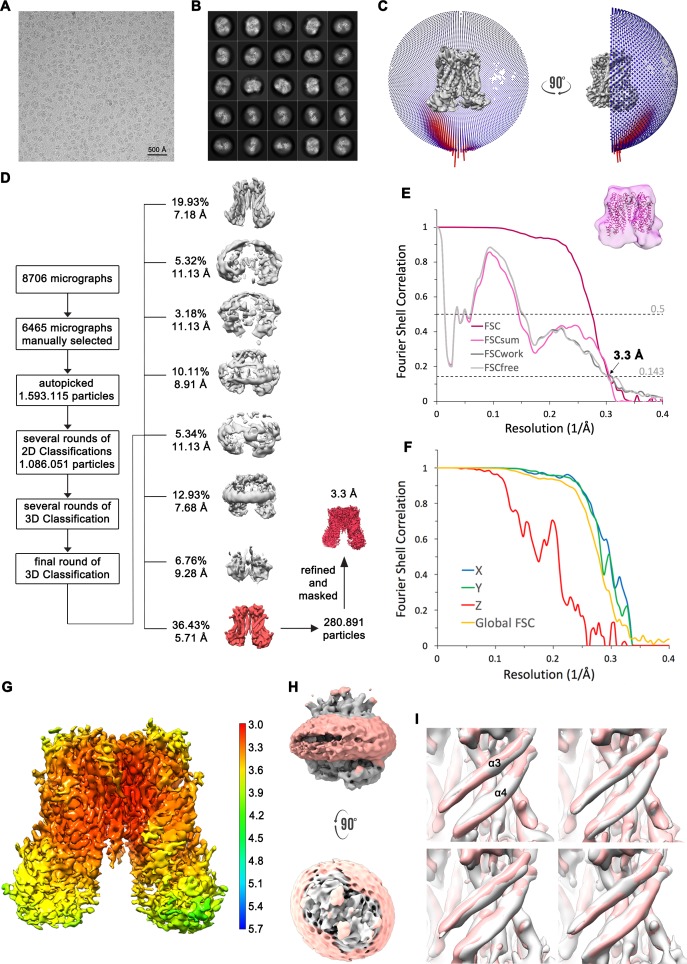
Structure determination of mTMEM16F in absence of Ca^2+^ in nanodiscs. (**A**) Representative cryo-EM image and (**B**) 2D-class averages of vitrified mTMEM16F in a Ca^2+^-free state in nanodiscs. (**C**) Angular distribution plot of particles included in the final C2-symmetric 3D reconstruction. The number of particles with their respective orientation is represented by length and color of the cylinders. (**D**) Image processing workflow. (**E**) FSC plot used for resolution estimation and model validation. The gold-standard FSC plot between two separately refined half-maps is shown in red and indicates a final resolution of 3.3 Å. FSC validation curves for FSC_sum_, FSC_work_ and FSC_free_, as described in Materials and methods, are shown as magenta, dark grey and light grey, respectively. A thumbnail of the mask used for FSC calculation overlaid on the atomic model is shown in the upper right corner and threshold used for FSC_sum_ of 0.5 and for FSC of 0.143 are shown as dashed lines. (**F**) Anisotropy estimation plot of the final map. The global FSC curve is represented in yellow. The directional FSCs along the x, y and z axis are shown in blue, green and red, respectively, reveal a strongly anisotropic dataset, which compromises a detailed interpretation of the map. (**G**) Final reconstruction map colored by local resolution as estimated by Relion. (**H**) Representation of the map including the nanodisc (low-pass filtered to 6.5 Å) reveals no noticeable distortion of the nanodisc (light red). (**I**) Analysis of structural heterogeneity, by subjecting the final dataset to another round of 3D classification, reveals no alternative conformations of α3 and α4. Shown are superpositions of obtained 3D classes (grey) on the final cryo-EM map (light red), low-pass filtered to 6.5 Å.

**Figure 2—figure supplement 5.**
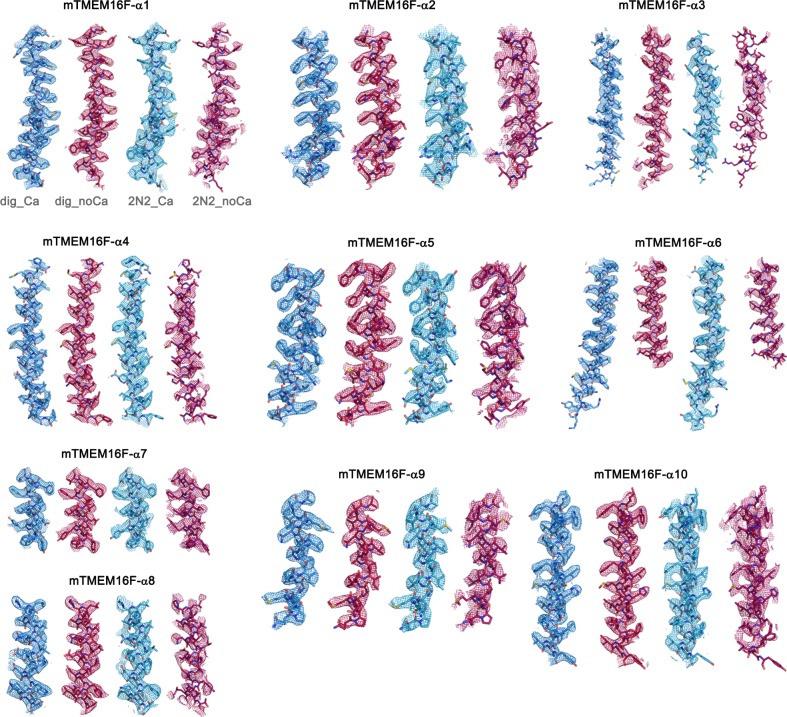
Cryo-EM Density Maps. Sections of the cryo-EM density of all four mTMEM16F maps superimposed on the respective refined models. Models are shown as sticks and structural elements are labelled. mTMEM16F Ca^2+^-bound in digitonin is colored in blue, mTMEM16F Ca^2+^-free in digitonin in red, mTMEM16F Ca^2+^-bound in nanodiscs in light blue and mTMEM16F Ca^2+^-free in nanodiscs in magenta. Maps were sharpened with a b-factor of –94, –139, –121 and –131 Å^2^ and contoured at 4.7 σ, 4.7 σ, 6.5 σ and 6.5 σ, respectively.

**Figure 2—figure supplement 6.**
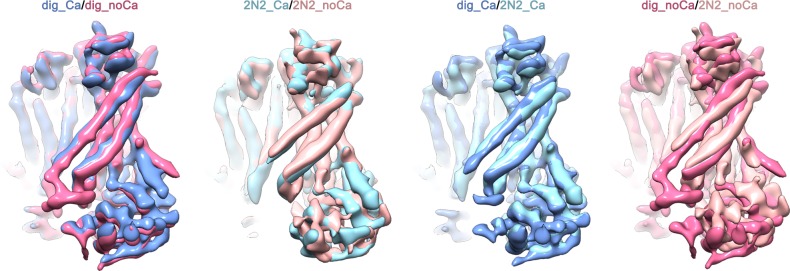
Superpositions of the four final cryo-EM maps. mTMEM16F in presence of calcium is shown in dark blue in digitonin and in light blue when reconstituted in nanodiscs. mTMEM16F in absence of calcium is shown in magenta in digitonin and in coral when reconstituted in nanodiscs. For a better comparison all maps were low-pass filtered to 6.5 Å. The conformational differences observed for α3 and α4 between the datasets obtained in digitonin and nanodiscs might hint towards a lipid-dependent conformational transition.

**Figure 2—figure supplement 7.**
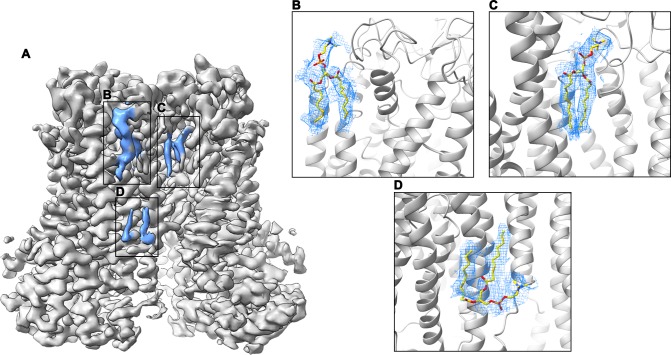
Cryo-EM density of lipids at the dimer interface. (**A**) Cryo-EM map of mTMEM16F in digitonin in the presence of Ca^2+^ (grey). Residual density at the dimer interface is highlighted in blue. (**B–D**) Close-up of putative lipid densities (blue mesh) with modelled phosphatidylcholine. The protein is shown as grey ribbon. The map was low-pass filtered to 6.5 Å.

**Table 1.** Cryo-EM data collection, refinement and validation statistics.

	mTMEM16F dig, +Ca^2+^ (EMDB 4611, PDBID 6QP6)	mTMEM16F dig, -Ca^2+^ (EMDB 4612, PDBID 6QPB)	mTMEM16F 2N2, +Ca^2+^ (EMDB 4613, PDBID 6QPC)	mTMEM16F 2N2, -Ca^2+^ (EMDB 4614, PDBID 6QPI)
Data collection and processing				
Microscope Camera Magnification	FEI Talos Arctica Gatan K2 Summit + GIF 49,407	FEI Talos Arctica Gatan K2 Summit + GIF 49,407	FEI Talos Arctica Gatan K2 Summit + GIF 49,407	FEI Talos Arctica Gatan K2 Summit + GIF 49,407
Voltage (kV) Exposure time frame/total (s) Number of frames per image	200 0.15/9 60	200 0.15/9 60	200 0.15/9 60	200 0.15/9 60
Electron exposure (e–/Å^2^)	52	52	52	52
Defocus range (μm)	−0.5 to −2.0	−0.5 to −2.0	−0.5 to −2.0	−0.5 to −2.0
Pixel size (Å) Box size (pixels)	1.012 220	1.012 220	1.012 256	1.012 220
Symmetry imposed	C2	C2	C2	C2
Initial particle images (no.)	1,348,247	1,314,676	1,019,012	1,593,115
Final particle images (no.)	219,302	194,284	186,487	280,891
Map resolution (Å) 0.143 FSC threshold	3.18	3.64	3.54	3.27
Map resolution range (Å)	2.9–4.5	3.2–5	3.3–5	3.0–4.2
				
Refinement				
Initial model used	PDBID 5OYB	PDBID 6QP6	PDBID 6QP6	PDBID 6QPB
Model resolution (Å) FSC threshold	3.3	3.8	4.0	7.0
Model resolution range (Å)	80–3.2	80–3.6	80–3.5	80–3.3
Map sharpening *B* factor (Å^2^)	−94	−139	−121	−131
Model composition Nonhydrogen atoms Protein residues Ligands	12144 1462 8	12136 1460 2	9060 1138 6	2288 462 -
*B* factors (Å^2^) Protein Ligand	53.3 39.1	52 34.8	72.7 54.6	7.8 -
R.m.s. deviations Bond lengths (Å) Bond angles (°)	0.009 1	0.007 0.9	0.005 0.87	0.004 0.655
Validation MolProbity score Clashscore Poor rotamers (%)	1.36 6.5 0.46	1.23 4.5 0.3	1.3 5.6 0.22	1.17 2.4 0
Ramachandran plot Favored (%) Allowed (%) Disallowed (%)	98.6 1.4 0	98.4 1.6 0	98.4 1.6 0	97.2 2.8 0

**Video 1.** Structure of mTMEM16F in complex with Ca^2+^. Shown is the cryo-EM density map of mTMEM16F obtained in complex with Ca^2+^ in digitonin superimposed on the refined structure. For clarity, only a single subunit is displayed. The cryo-EM map is depicted as blue surface, the model as blue sticks, and calcium ions as green spheres.

**Video 2.** Structure of mTMEM16F in absence of Ca^2+^. Shown is the cryo-EM density map of the mTMEM16F obtained in a Ca^2+^-free state in digitonin superimposed on the refined structure. For clarity, only a single subunit is displayed. The cryo-EM map is depicted as magenta surface and the model as red sticks.

To investigate the effect of a lipid bilayer on the structural properties of mTMEM16F, we reconstituted the protein into large 2N2 lipid nanodiscs with a lipid composition that retains scrambling activity, although with slower kinetics (Figure 1—figure supplement 1D), and collected cryo-EM data of both states in a membrane-like environment (Figure 2—figure supplements 3 and 4). While of similar nominal resolution to the data obtained in detergent, the preferred orientation of the protein in nanodiscs on the grids resulted in anisotropic cryo-EM maps, in which some of the details are blurred (Figure 2—figure supplements 3–5). This problem is less pronounced in the data obtained in nanodiscs in presence of Ca^2+^ than in the dataset of the Ca^2+^-free sample. Nonetheless, both datasets are of sufficient quality to allow for a comparison of corresponding states of mTMEM16F in a detergent and lipid environment. For most parts, these structures are virtually indistinguishable except for α3 and α4, which show a larger tilt towards α6 in the cryo-EM maps of nanodisc samples, a feature that is more prominent in the Ca^2+^-free state (Figure 2—figure supplement 6). Thus, our data reveal a general equivalence of structures in detergent and in a membrane environment but they also hint at context-dependent differences in a region that constitutes the ion and lipid permeation path (Figure 2—figure supplements 5 and 6). In addition, we were interested whether we could identify any structural flexibility within each of the four datasets. While no heterogeneity was found in the data in absence of Ca^2+^ both in detergent and nanodiscs (Figure 2—figure supplements 2I and 4I), we were able to identify classes with slightly altered conformations for α3 and α4 in the detergent and nanodiscs samples in presence of Ca^2+^ (Figure 2—figure supplements 1I and 3I). These differences are more pronounced in the Ca^2+^-bound nanodisc sample and could point towards a further structural transition of these α-helices from their current position into an open conformation of the ‘subunit cavity’, as observed for the fungal scramblases nhTMEM16 (Kalienkova et al., 2019) and afTMEM16 (Falzone et al., 2019) and for the human TMEM16K (hTMEM16K, Bushell et al., 2018). However, despite the conformational spread of α3 and α4, no class showing an open conformation of the ‘subunit cavity’ was identified in our data (Figure 2—figure supplement 6).

Unassigned densities near the dimer interface present in all cryo-EM maps most likely account for tightly bound lipids (Figure 2—figure supplement 7). Although the potential functional relevance of these lipids is still unclear, similar densities were found in other mammalian TMEM16 proteins, such as mTMEM16A (Paulino et al., 2017a; Dang et al., 2017) and hTMEM16K (Bushell et al., 2018), but not in fungal homologs. In contrast to mTMEM16A (Paulino et al., 2017b), nhTMEM16 (Kalienkova et al., 2019) and afTMEM16 (Falzone et al., 2019) no obvious distortion of the detergent micelle or the lipid nanodiscs was observed in the maps of mTMEM16F (Figure 2—figure supplements 1H, 2H, 3H and 4H). Yet, in light of the structural similarity between family members, a destabilizing effect of the protein in its active conformation on the membrane is conceivable. Due to the superior quality of the structures of mTMEM16F in detergent, they are used for further structural analysis.

### Relationship to other TMEM16 structures

**Figure 3.**
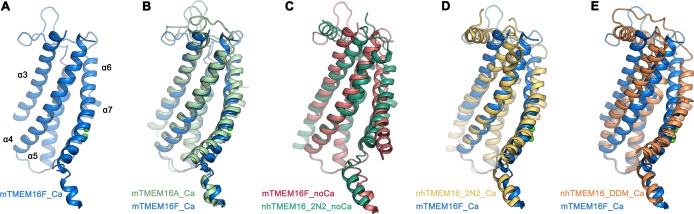
Comparison of the ‘subunit cavity’ of TMEM16 homologues. (**A**) ‘Subunit cavity’ in the Ca^2+^-bound mTMEM16F structure composed of α-helices 3–7 (blue). (**B**) Superposition of the ‘subunit cavity’ between the Ca^2+^-bound structures of mTMEM16F (blue) and the anion channel mTMEM16A (PDBID 5OYB (Paulino et al., 2017a), green). (**C**) Superposition of the ‘subunit cavity’ between the Ca^2+^-free structure of mTMEM16F (magenta) and the Ca^2+^-free structure of the fungal scramblase nhTMEM16 in nanodiscs (PDBID 6QM4 (Kalienkova et al., 2019), teal) displaying a closed state of the scramblase. (**D**) Superposition of the Ca^2+^-bound structure of mTMEM16F (blue) with a Ca^2+^-bound conformation of nhTMEM16 in nanodiscs (PDBID 6QMA (Kalienkova et al., 2019), yellow) displaying an intermediate state of the scramblase. (**E**) Superposition of the Ca^2+^-bound structure of mTMEM16F (blue) with a Ca^2+^-bound conformation of nhTMEM16 in detergents (PDBID 6QM5 (Kalienkova et al., 2019), orange) displaying an open state of the scramblase.

With a pairwise sequence identity of 38%, mTMEM16F is closer to the anion channel mTMEM16A (Figure 3—figure supplement 1) than to the more distantly related fungal scramblase nhTMEM16 and its mammalian orthologue hTMEM16K, with which it shares sequence identities of 21% and 25%, respectively. It is thus not surprising that, with respect to its general architecture, mTMEM16F closely resembles mTMEM16A (Figure 3—figure supplement 2). The similarity to mTMEM16A extends to all parts of the protein, including the cytoplasmic and extracellular domains, and it is particularly pronounced in the Ca^2+^-bound conformations, which superimpose with a root mean square deviation (RMSD) of 2.1 Å (Figure 3—figure supplement 2A). The RMSD decreases to 1 Å when relating Cα-positions of the transmembrane domain of a single subunit (encompassing 298 Cα-positions, which are also used in superpositions with nhTMEM16, Figure 3A,B). The potential correspondence of both structures of mTMEM16F to functional states of a scramblase is best illustrated in their comparison to nhTMEM16, for which closed, intermediate and open conformations have been determined (Kalienkova et al., 2019). The equivalence of the Ca^2+^-free conformation of mTMEM16F with an inactive ligand-free state of a TMEM16 scramblase is evident in the low RMSD of 2.0 Å of a superposition with the Ca^2+^-free conformation of nhTMEM16 in nanodiscs (Figure 3C). In the case of Ca^2+^-bound structures, the relationship of mTMEM16F to the intermediate conformation of nhTMEM16 observed in nanodiscs is, with an RMSD of 1.9 Å, much closer than to the open conformation of the same protein, where the RMSD of 3.1 Å underlines their nonequivalence (Figure 3D,E; Figure 3—figure supplement 2B). The large differences originate from the diversity of conformations of the subunit cavity observed for Ca^2+^-bound states of TMEM16 proteins. The cavity is accessible to the membrane in the case of the Ca^2+^-bound open state of nhTMEM16 (Brunner et al., 2014; Kalienkova et al., 2019) and equivalent conformations of its close homologues afTMEM16 (Falzone et al., 2019) and hTMEM16K (Bushell et al., 2018), but not in the Ca^2+^-bound intermediate and closed conformations of nhTMEM16 (Kalienkova et al., 2019) and the observed Ca^2+^-bound conformation of mTMEM16F (Figure 3C–E). It is thus uncertain whether the here captured Ca^2+^-bound structure of mTMEM16F shows a partly activated state, which is incapable of scrambling lipids, akin to the intermediate state of nhTMEM16, or whether the scrambling mechanism of mTMEM16F differs from nhTMEM16 and its mammalian orthologues. In light of the ion permeation properties of mTMEM16F and its similarity to mTMEM16A, it is conceivable that the here observed Ca^2+^-bound structure is close to a conducting conformation for ions. In that respect, the question is pertinent why mTMEM16F but not mTMEM16A catalyzes lipid movement.

**Figure 3—figure supplement 1.**
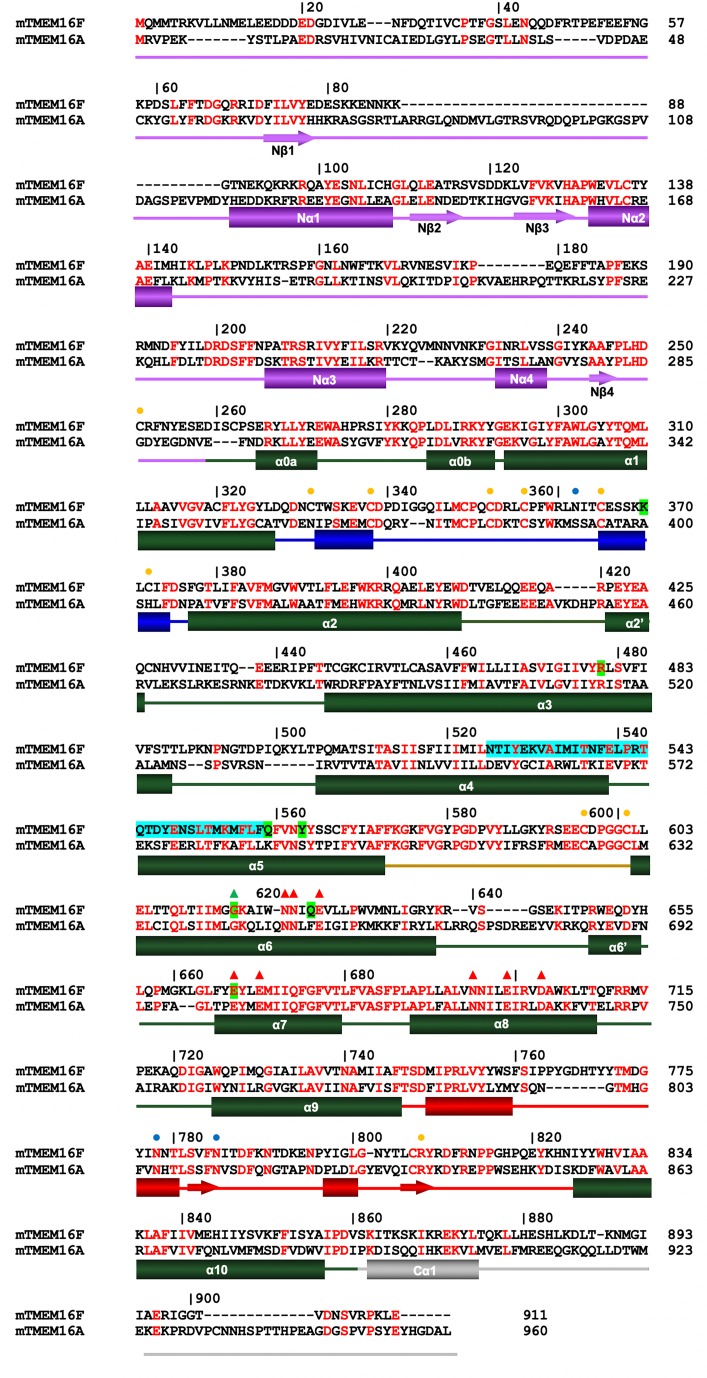
Sequence alignment. Protein sequences of murine TMEM16F (UniProt: Q6P9J9.1) and murine TMEM16A (UniProt: Q8BHY3.2) were aligned with Clustal Omega (Sievers et al., 2011). Numbering corresponds to mTMEM16F. Secondary structure elements: green, transmembrane domain; violet, N-terminal domain; grey, C-terminal region; blue, α1–α2 loop; beige, α5–α6 loop; red, α9–α10 loop. Boundary of secondary structure elements refers to mTMEM16F. Orange circles mark the position of cysteines involved in disulfide bonds, blue circles mark putative sites of glycosylation indicated by residual density in the cryo-EM map. Red triangles correspond to residues of the Ca^2+^-binding site and the green triangle to the hinge for conformational rearrangements in α6. The SCRD is highlighted in cyan and positions mutated in this study in green.

**Figure 3—figure supplement 2.**
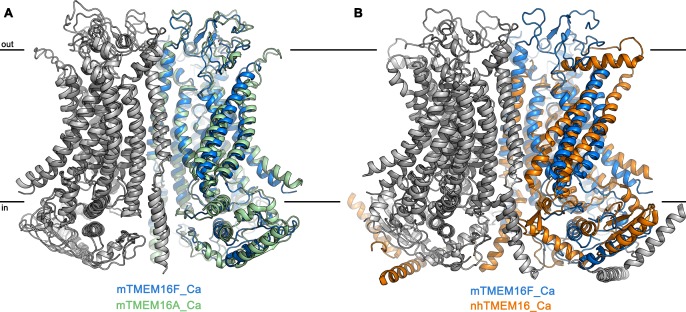
Superposition of TMEM16 dimers. (**A**) Superposition of the Ca^2+^-bound structures of mTMEM16F in digitonin (blue and grey) and the anion channel mTMEM16A (PDBID 5OYB (Paulino et al., 2017a), green and grey). (**B**) Superposition of the Ca^2+^-bound structures of mTMEM16F (blue and grey) and the fungal scramblase nhTMEM16 (PDBID 6QM5 (Kalienkova et al., 2019), orange and grey). A, B, The view is as in Figure 2C.

### Architecture of the ‘subunit cavity’

**Figure 4.**
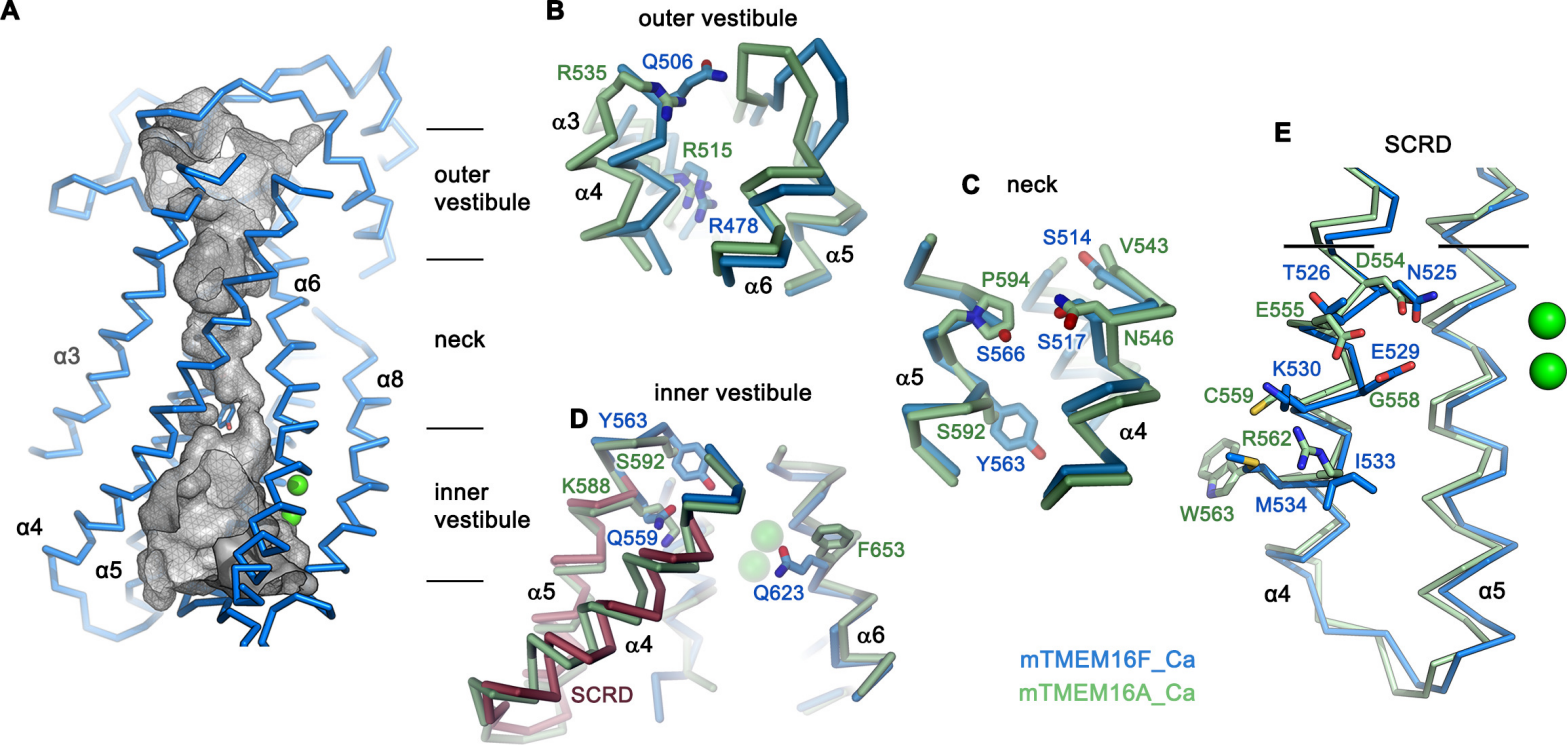
Pore region of mTMEM16F. (**A**) ‘Subunit cavity’ in the Ca^2+^-bound state of mTMEM16F showing the putative ion conduction pore. Grey mesh shows pore surface (sampled with probe radius of 1 Å). (**B** to **D**) Superposition of selected pore regions of mTMEM16F (blue) and the anion channel mTMEM16A (green, remodeled version of PDBID 5OYB, see Materials and methods) along the extracellular vestibule (**B**), the neck (**C**) and the intracellular vestibule, with the 35 amino acid long scrambling domain (SCRD) on α4 and α5 of mTMEM16F highlighted in brown (**D**). (**E**) View of the SCRD rotated by about 90° relative to D. The SCRD boundary is indicated by black lines. A-E, Selected residues are displayed as sticks, bound calcium ions as green spheres.

The structure of the putative catalytic unit of mTMEM16F in a Ca^2+^-bound conformation shows a strong resemblance to the ion permeation path in mTMEM16A, where an hourglass-shaped pore contains two aqueous vestibules on either side of the membrane that are bridged by a narrow neck (Paulino et al., 2017a) (Figure 4—figure supplement 1A). A related architecture is observed in mTMEM16F, where closer interactions between α-helices and the presence of bulky residues along the path further constrict the pore below the size of permeating ions (Figure 4A–E; Figure 4—figure supplement 1A,B). Thus, it is unclear whether the observed conformation of mTMEM16F could facilitate ion conduction. In mTMEM16A, a strong positive electrostatic potential throughout the pore, originating from an excess of cationic residues, determines its selectivity for anions (Paulino et al., 2017a). Due to an altered distribution of charges in the pore region of mTMEM16F, the potential is less positive on both entrances to the narrow neck, which is consistent with the poor selectivity of currents passed by this protein (Figure 4—figure supplement 1C; Figure 1—figure supplement 1I).

**Figure 4—figure supplement 1.**
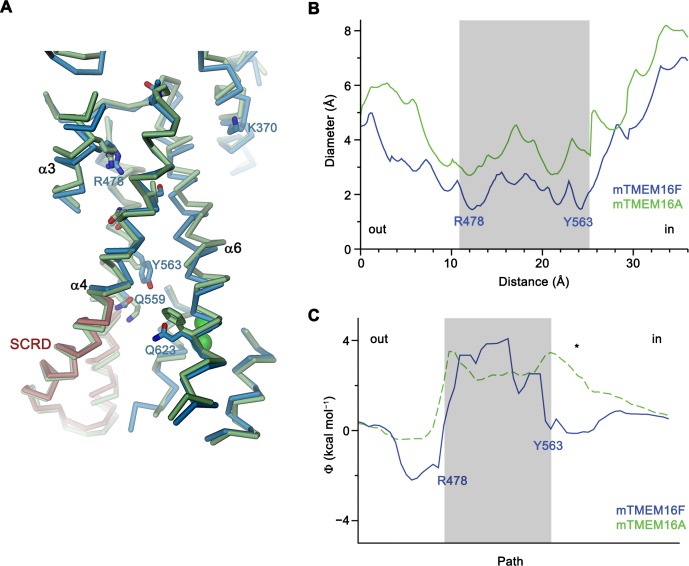
Structural features of the pore region and electrostatics. (**A**) View of the catalytic regions in a superposition of mTMEM16F (blue) and mTMEM16A (green, remodeled version of PDBID 5OYB, see Materials and methods). The SCRD is colored in brown. Selected residues are displayed as sticks. Labels and numbers correspond to mTMEM16F. (**B**) Diameter of the putative pores of mTMEM16F (blue) and mTMEM16A (green) as calculated by HOLE (Smart et al., 1996) from the extracellular (out) to the intracellular side (in). The positions of Arg 478 and Tyr 563 are indicated. (**C**) Electrostatic profile calculated along the pore regions of mTMEM16F (blue) and mTMEM16A (green, dashed, obtained from Paulino et al., 2017a). The potential is less positive in mTMEM16F for most of the pore, consistent with the observed slight preference for cations over anions. Due to its narrow diameter, the potential in the neck is less accurate. * indicates location of the Ca^2+^-binding site. B,C, The positions of Arg 478 and Tyr 563 are indicated and the region of the neck is highlighted in grey.

Whereas in the extracellular vestibule the uncharged Gln 506, which has replaced an arginine at the equivalent position in mTMEM16A, lowers the positive charge density in the outer mouth of the pore, Arg 478 at the extracellular boundary to the neck is conserved (Figure 4B). The neck of mTMEM16F exhibits a similar amphiphilic character as found in mTMEM16A but it contains several changes, which decrease its hydrophobicity (Figure 4C). Further towards the cytoplasm, the bulky Tyr 563 lowers the pore diameter of mTMEM16F to 1.6 Å (Figure 4 C,D; Figure 4—figure supplement 1B). Finally, at the boundary to the wide intracellular vestibule, Gln 559 replaces a lysine at the equivalent position in mTMEM16A, thereby removing another positive charge from the putative ion permeation path (Figure 4D).

Similar to its paralogue mTMEM16A, the interacting α-helices 4 and 6 disengage towards the cytoplasm and open a hydrophilic gap to the membrane, which causes a dilation of the pore at the intracellular vestibule (Figure 4D). In mTMEM16F, this region contains the scrambling domain (SCRD), encompassing the intracellular parts of α4 and α5, which was previously described as determinant for lipid movement (Gyobu et al., 2017; Yu et al., 2015) (Figure 4D,E). The intracellular gap of mTMEM16F also parallels the equivalent region of nhTMEM16, but the closure of the cavity towards the extracellular side interrupts the polar and membrane-accessible furrow found in the Ca^2+^-bound ‘open state’ of the fungal scramblase nhTMEM16 and instead resembles its Ca^2+^-bound intermediate observed in nanodiscs (Kalienkova et al., 2019) (Figure 3D,E). The region of the closed cavity that faces the lipid bilayer in mTMEM16F is hydrophobic and resembles mTMEM16A, except for few residues such as Gln 623, located on α6 facing the SCRD, and Lys 370 located at the extracellular domain close to the membrane boundary (Figure 4D; Figure 4—figure supplement 1A).

### Calcium activation

**Figure 5.**
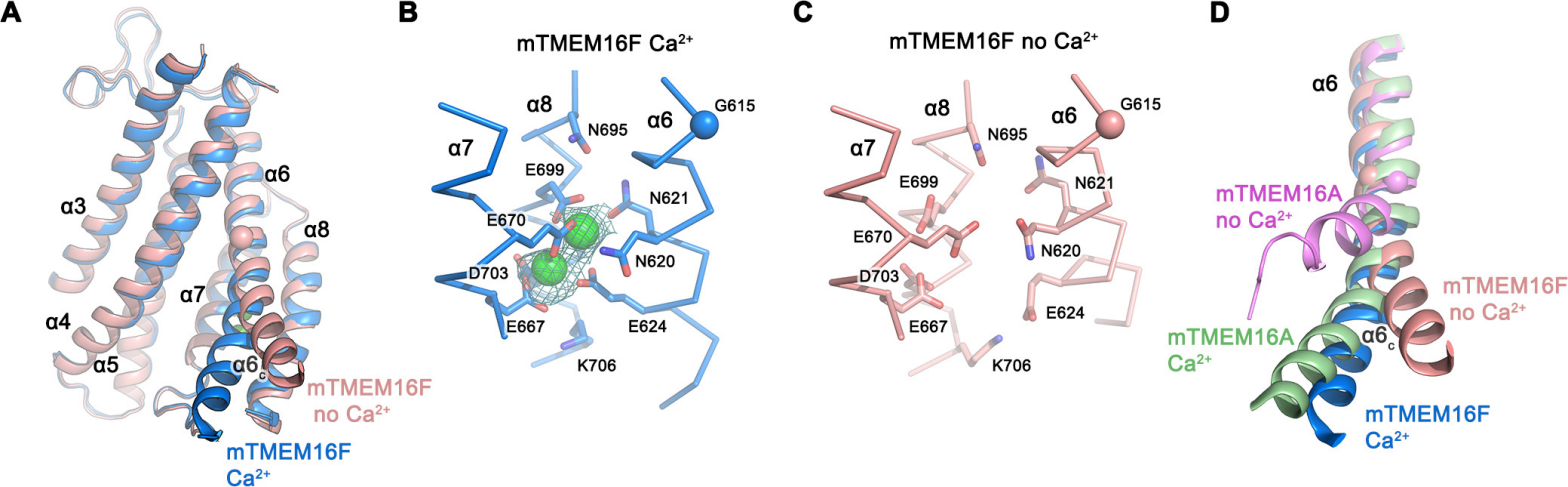
Ca^2+^-binding site and conformational changes. (**A**) Superposition of α-helices 3–8 of mTMEM16F in the presence (blue) and absence of Ca^2+^ (coral). (**B and C**) Close-up of the Ca^2+^-binding site in complex (**B**) or in absence of Ca^2+^ (**C**). Coordinating residues are depicted as sticks, calcium ions as green spheres (with surrounding cryo-EM density contoured at 5σ). (**D**) Comparison of Ca^2+^-induced movements in mTMEM16F (Ca^2+^-bound in blue, Ca^2+^-free in coral) and mTMEM16A (Ca^2+^-bound (PDBID 5OYB) in green, Ca^2+^-free (PDBID 5OYG) in pink). Shown is a cartoon superposition of α6. A-D. Pivot for movement is depicted as sphere.

The structure of mTMEM16F in the presence of Ca^2+^ defines the location of two Ca^2+^ ions in each subunit that are bound to a site which strongly resembles its counterpart in nhTMEM16 (Brunner et al., 2014) and mTMEM16A (Paulino et al., 2017a) (Figure 5; Figure 5—figure supplement 1A). In this site, conserved acidic and polar residues located on α-helices 6–8 coordinate the ions with similar geometry. Different from its paralogue functioning as anion channel, mTMEM16F does not contain an insertion of a residue in α6 near the hinge (Figure 3—figure supplement 1), which causes a partial unwinding and the formation of a π-bulge in mTMEM16A (Paulino et al., 2017a). Consequently, the helix in the ligand-bound conformation of mTMEM16F might be in a less strained conformation.

**Figure 5—figure supplement 1.**
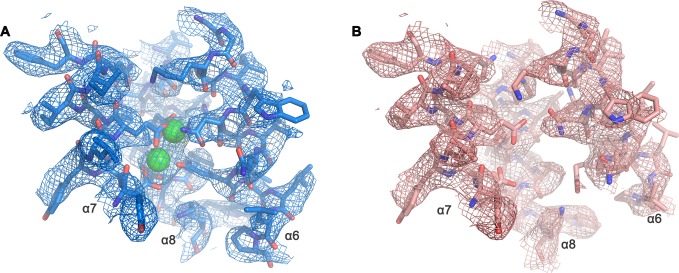
Cryo-EM density of the Ca^2+^-binding site. Close-up of Ca^2+^-binding site of the Ca^2+^-bound (**A**) and Ca^2+^-free (**B**) state of mTMEM16F in digitonin as depicted in Figure 5B. A, B, The respective cryo-EM density is shown as mesh, all residues are displayed as sticks and Ca^2+^ as green spheres. Maps were sharpened with a b-factor of –94,–139 Å^2^, respectively, and both contoured at 5 σ.

**Figure 5—figure supplement 2.**
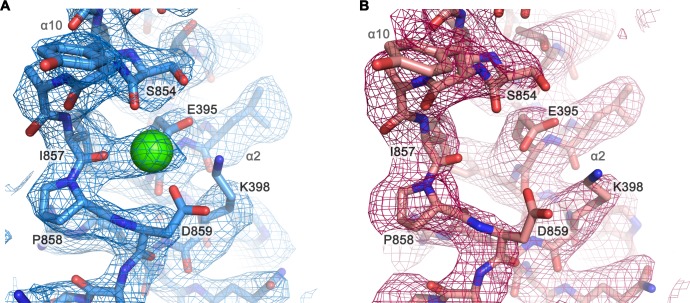
Cryo-EM density of a putative Ca^2+^-binding site located at the end of α10. Close-up of a putative Ca^2+^-binding site in the Ca^2+^-bound state of mTMEM16F in digitonin (**A**) and in the Ca^2+^-free state of mTMEM16F in digitonin (**B**). The respective cryo-EM densities are shown as mesh, the models as sticks and Ca^2+^ as green sphere. Selected residues are labeled. Maps were contoured at 4.2 σ and sharpened with a b-factor of –94,–139 Å^2^, respectively.

The conformational changes that take place in mTMEM16F upon ligand binding can be appreciated in the comparison of Ca^2+^-bound and Ca^2+^-free conformations (Figures 2C and 5A–C; Figure 5—figure supplement 1). As in mTMEM16A, the absence of Ca^2+^ causes a local transition confined to the pore region, whereas the remainder of the structure remains largely unaffected (Figures 2C and 5A). Besides comparably small rearrangements of α3 and α4, which are less pronounced in the nanodisc structures (Figure 2—figure supplement 6), the largest difference is found at the intracellular half of α6 (α6_c_, Figure 5A). In the presence of Ca^2+^, the bound ligands provide an interaction platform for negatively charged and polar residues on α6, thereby immobilizing the helix in the observed conformation (Figure 5B). In the ligand-free state, this helix has altered its conformation in response to the loss of the positively charged interaction partner, leading to an increase in mobility of α6_c_ as indicated by the weaker density (Figure 5—figure supplement 1B). The transition can be described by a rigid-body movement of α6_c_ by 20°, around a hinge located close to a conserved glycine residue (Gly 615, Figure 3—figure supplement 1), whose equivalent in mTMEM16A was shown to be critical for conformational rearrangements (Paulino et al., 2017a). However, in contrast to mTMEM16A, the observed change in mTMEM16F is considerably smaller, proceeds in the opposite direction and it is not accompanied by a rotation around the helix axis (Figure 5D). Thus, whereas in the apo-form of mTMEM16A α6_c_ approaches α4, thereby closing the gap between both helices, the movement of α6_c_ in mTMEM16F is directed away from α4 (Figure 5A,D). The observed structural transition opens the access of the ion binding site to the cytoplasm and widens the gap to the SCRD, which likely contributes to the inhibition of scrambling by increasing the energy barrier for lipid flipping by a currently unclear mechanism.

In the recent structure of hTMEM16K, which was crystallized in 100 mM Ca^2+^ (Bushell et al., 2018), besides the two Ca^2+^ in the consensus site responsible for activation, an additional Ca^2+^ ion was identified to bind to a site at the intracellular end of α10. This ion is coordinated by negatively charged residues located on α2 and the loop following α10, which are conserved among mammalian but not fungal homologues. Residual density in the Ca^2+^-containing data, which is absent in data of Ca^2+^-free samples, indicate the presence of an ion at the equivalent position in mTMEM16F (Figure 5—figure supplement 2). A similar unassigned density is also found in the Ca^2+^-containing data of mTMEM16A (Paulino et al., 2017b), but not in fungal homologues (Falzone et al., 2019; Kalienkova et al., 2019), thus revealing a position of still unclear functional relevance that is shared among mammalian TMEM16 family members and can be occupied by Ca^2+^ at μM concentrations.

### Functional characterization of mutants of the Ca^2+^-binding site

Whereas the described structures revealed the detailed architecture of mTMEM16F and its conformational changes in response to ligand binding, the similarity of the protein to its paralogue working as anion channel leaves important aspects of structure-function relationships ambiguous.

**Figure 6.**
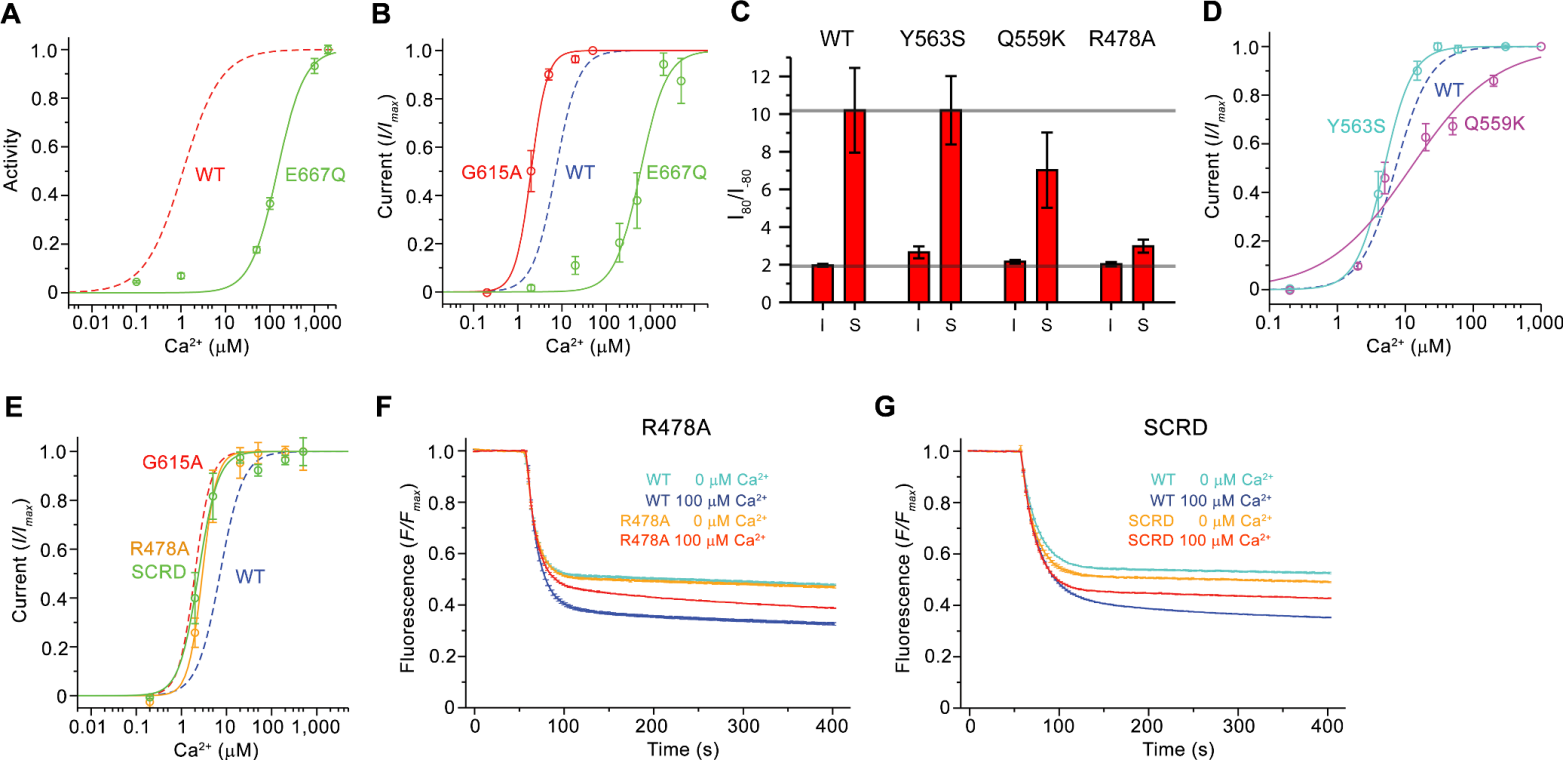
Functional properties of mutants. (**A**) Ca^2+^ concentration-response relationship of lipid scrambling in the binding site mutant E667Q. Data show average of either six (0, 1, 10, 100, 1000 µM) or three (other concentrations) independent experiments from two protein reconstitutions. (**B**) Ca^2+^ concentration-response relationship of currents of E667Q (n = 4) and the hinge residue G615A (n = 4). (**A and B**) For comparison WT traces are shown as dashed lines. (**C**) Rectification indices (I_80mV_/I_–80mV_) of instantaneous (I) and steady-state currents (S) of WT (n = 12) and the mutants Y563S (n = 8), Q559K (n = 10) and R478A (n = 10). Unlike Q599K, the difference between R478A and WT was found to be statistically significant. (**D and E**) Ca^2+^ concentration-response relationship of currents of mutants Y563S (n = 3) and Q559K (n = 5) (**D**) and R478A (n = 5) and mTMEM16F^SCRD^ (SCRD, n = 6) (**E**). (**F and G**) Scrambling of the mutants R478A (**F**) and mTMEM16F^SCRD^ (SCRD) (**G**). F, G Data show averages of three independent experiments of one protein reconstitution. WT reconstituted in a separate set of liposomes from the same batch is shown in comparison. B, D, E Data show averages from independent biological replicates. A-G, Errors are s.e.m.

The first question we have addressed was whether Ca^2+^-binding to the conserved site of mTMEM16F would affect scrambling and ion conduction in a similar manner. We have thus investigated the scrambling activity of mutants of the Ca^2+^-binding site and found a strong decrease in the potency of Ca^2+^ in the mutant E667Q located on α7 and little detectable activity in the mutant E624Q on α6 (Figures 5B and 6A; Figure 6—figure supplement 1A,B), consistent with a very similar phenotype of equivalent mutants on the activation of the chloride channel mTMEM16A (Brunner et al., 2014; Lim et al., 2016; Tien et al., 2014; Yu et al., 2012). By using patch-clamp electrophysiology, we found a comparable effect of the same mutations on the activation of currents, in line with previous observations (Yang et al., 2012), thus emphasizing the mutuality in the regulation of both processes (Figures 5B and 6B; Figure 6—figure supplement 1C,D). We next investigated the role of the observed conformational change of α6 for activation and the importance of the conserved glycine position as hinge for its movement. In patch-clamp experiments, the mutation of the conserved Gly 615 to alanine increased the potency of Ca^2+^ (Figure 6B; Figure 6—figure supplement 1E), indicating a stabilization of the open state upon mutation of this flexible position, which parallels a behavior observed in the channel mTMEM16A (Paulino et al., 2017a; Peters et al., 2018). Hence, the effect of mutations on the Ca^2+^-binding site and the gating hinge indicate that the conformational changes observed in α6 are the basis of a common mechanism of activation of ion conduction and scrambling in mTMEM16F.

**Figure 6—figure supplement 1.**
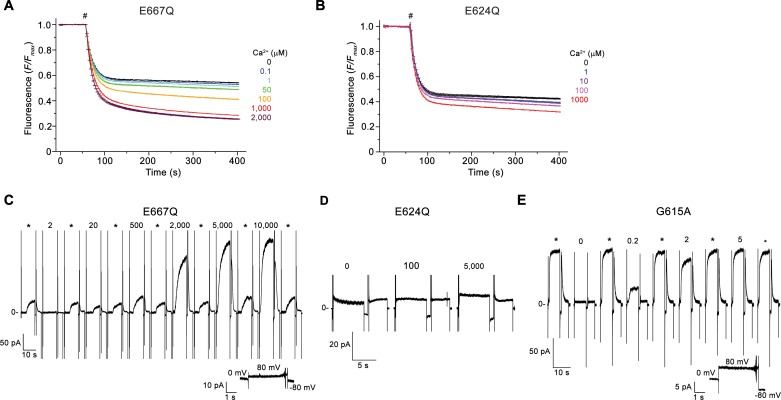
Electrophysiology and lipid scrambling data of mutants of the Ca^2+^-binding site and the gating hinge. (**A and B**) Ca^2+^-dependence of scrambling activity in proteoliposomes containing the Ca^2+^-binding site mutants E667Q (**A**) and E624Q (**B**) at indicated ligand concentrations. (**C, D and E**) Representative current response of the mTMEM16F mutants E667Q (**C**), E624Q (**D**), and G615A (**E**) at different Ca^2+^ concentrations. C-E Data were recorded from inside-out patches excised from HEK293 cells transfected with mTMEM16F constructs fused to fluorescent proteins. Numbers indicate Ca^2+^ concentrations (µM), * indicates reference pulses used for rundown correction recorded at 200 µM Ca^2+^ for E667Q and 20 µM Ca^2+^ for all other mutants. A, B Traces depict fluorescence decrease of tail-labeled NBD-PE lipids after addition of dithionite (#). Data show averages of three technical replicates from the same reconstitution. C, E, Inset (bottom) shows leak currents at applied voltages in the absence of Ca^2+^.

**Figure 6—figure supplement 2.**
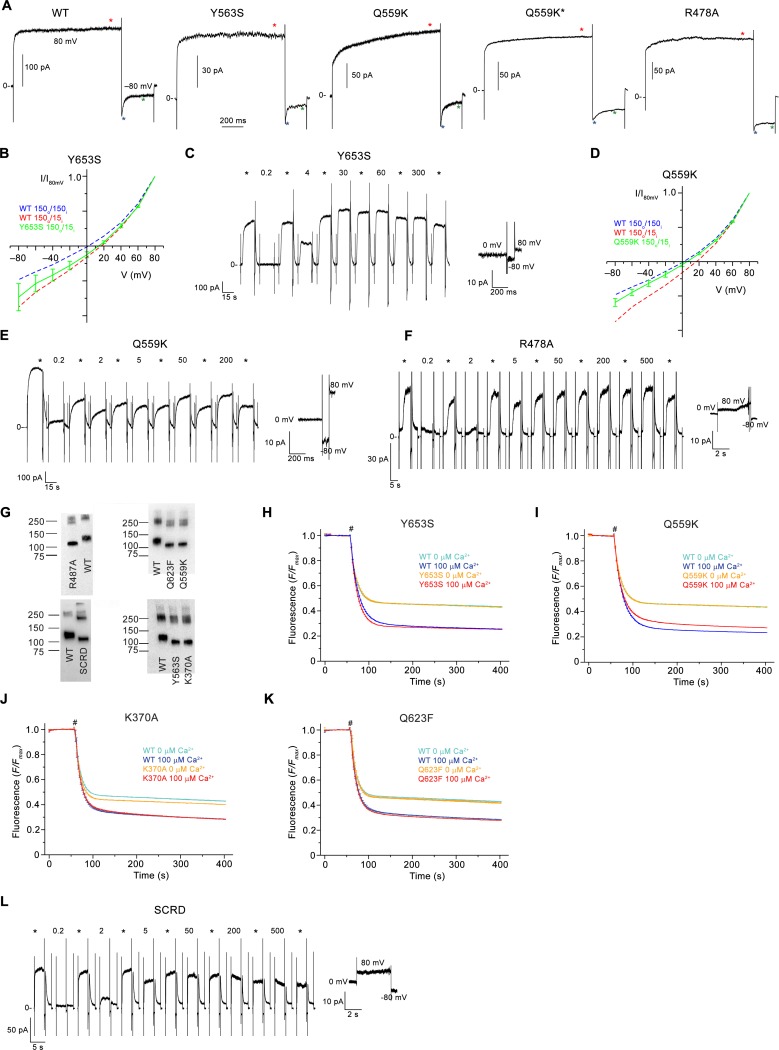
Electrophysiology and lipid scrambling data of mutants of the pore region. (**A**) Representative current traces of WT and mutants used for calculation of the rectification index (I_80mV_/I_–80mV_) of instantaneous currents (6 ms after the voltage change) and at steady-state. Red (I_80mV_) and blue (I-_80mV_) asterisks mark the timepoints used for the calculation of rectification indices from instantaneous currents, green (I-_80mV_) the timepoint used for the calculation of rectification indices from steady-state currents. Q559K* corresponds to a subset of samples of the mutant with lower steady-state rectification. (**B**) Normalized I-V relationships of the mutant Y653S in a 10-fold gradient of NaCl (green). (**C**) Representative current response of the mTMEM16F mutant Y653S at different Ca^2+^ concentrations. (**D**) Normalized I-V relationships of the mutant Q559K in a 10-fold gradient of NaCl (green). The shift in the reversal potential in asymmetric conditions towards 0 indicates a decreased cation over anion selectivity. B,D, The osmolarity of low NaCl solution was adjusted by addition of NMDG-sulfate. WT at symmetric and asymmetric concentrations is indicated as dashed line for comparison. Data shows average of 3 (Y365S) and 5 (Q559K) biological replicates, errors are s.e.m.. (**E and F**) Representative current responses of the mTMEM16F mutants Q559K (**E**) and R478A (**F**) at different Ca^2+^ concentrations. (**G**) Western blot analysis of the protein content of proteoliposomes containing different mTMEM16F constructs after extraction with dodecyl-β-D-maltopyranoside. WT reconstituted in the same batch of liposomes as respective mutants was used as reference and is displayed on the same blot for comparison. Proteoliposomes of mutants show similar protein levels as respective WT controls and thus allow for a direct comparison of respective activities. (**H–K**) Scrambling activity of the mutants Y653S (**H**), Q559K (**I**) K370A (**J**) and Q623F (**K**) at indicated Ca^2+^ concentrations. (**L**) Representative current response of the construct mTMEM16F^SCRD^ (SCRD) at different Ca^2+^ concentrations. A-F, L, Data were recorded from inside-out patches excised from HEK293 cells transfected with mTMEM16F constructs fused to fluorescent proteins. Numbers indicate Ca^2+^ concentrations (µM), * indicates reference pulses used for rundown correction recorded at 20 µM Ca^2+^. C, E, F, L, Inset (bottom) shows leak currents at applied voltages in the absence of Ca^2+^. H-K, WT reconstituted in a separate set of liposomes from the same batch is shown as comparison. Traces depict fluorescence decrease of tail-labeled NBD-PE lipids after addition of dithionite (#). Data show averages of three technical replicates from the same reconstitution.

### Functional characterization of mutants affecting ion conduction and scrambling

After the characterization of the effect of mutations in the Ca^2+^-binding site on activation, we were interested in other structural elements mediating ion conduction and scrambling. The resemblance of the pore region of mTMEM16F and mTMEM16A suggests that ion conduction could proceed at the same location after local conformational changes that increase its diameter. We have thus investigated the influence of mutations of pore-lining residues on ion conduction and gating by analyzing the Ca^2+^ concentration-response relationships, ion selectivity and the rectification properties of currents. In all mutants, we either truncated the residues to alanine or replaced them with their equivalent amino acid found in mTMEM16A. Since, in the cryo-EM structure, Tyr 563 constricts the pore of mTMEM16F (Figure 4A,C; Figure 4—figure supplement 1A,B), we investigated the mutant Y563S. A similar Ca^2+^ potency and ion selectivity as the WT and no noticeable alteration of the rectification properties of currents were found (Figure 6C,D; Figure 6—figure supplement 2A–C), thus suggesting that Tyr 563 does not impose a rate-limiting barrier to ion conduction in the open state.

As described previously (Yang et al., 2012), the mutation of Gln 559 to lysine at the boundary between the intracellular vestibule and the narrow neck (Figure 4D) decreases the weak cation over anion selectivity of mTMEM16F, as expected for the introduction of a positively charged residue interacting with permeating ions (Figure 6—figure supplement 2D). In the mutant Q559K, we find a slightly lower Ca^2+^ potency and a markedly decreased slope in the Ca^2+^ concentration-response relationship compared to WT, as well as a moderate decrease in the rectification of steady-state currents, an effect that is particularly pronounced in a subset of the data (Figure 6C,D; Figure 6—figure supplement 2A,E). In contrast to the subtle phenotype of Y563S, the mutation of the conserved Arg 478 (Figure 4B) at the extracellular entrance of the narrow neck to alanine, yields a several-fold decreased current density compared to WT, yet with increased potency of Ca^2+^ and strongly decreased voltage-dependent relaxations. Consequently, the strong rectification at steady-state disappears, suggesting that the residue plays an important role during channel activation (Figure 6E; Figure 6—figure supplement 2A,F). Collectively, the observed changes in the activation and conduction properties of the mutants Q559K and R478A are consistent with their location in the pore. The increase of Ca^2+^-potency found in R478A and the strongly decreased time-dependent reduction of currents at negative voltage might indicate a stabilization of the open state. Moreover, the change in selectivity observed for the mutant Q559K provides direct evidence for its location at the pore lining.

As the mutation R478A at the extracellular part of the pore affected channel properties, we investigated its effect on lipid scrambling. Strikingly, this mutant showed a strongly decreased rate of lipid permeation (Figure 6F; Figure 6—figure supplement 2G), consistent with data previously obtained from cellular assays (Gyobu et al., 2017), whereas the activity of mutants Y563S and Q559K was robust (Figure 6—figure supplement 2G–I). Next, we investigated the scrambling activity of the mutants K370A and Q623F, which change the properties of two polar residues facing the outside of the subunit cavity (Figure 4B, Figure 4—figure supplement 1A). Both residues are located at opposite ends of the otherwise hydrophobic surface of the contact region between α4 and α6. They were selected under the assumption that mutations would interfere with lipid transport in a mechanism that does not rely on the opening of the subunit cavity. Conversely, they would be located on the outside and thus not interfere with lipid scrambling in a mechanism that requires cavity opening. Since both mutants did not show a detectable effect on scrambling, they are likely not located on the lipid permeation path (Figure 6—figure supplement 2G,J,K). By contrast, the strong inhibitory effect of the mutation R478A, suggests that an opening of the subunit cavity is necessary to bring this otherwise buried residue into direct contact with permeating lipids.

Finally, we searched for additional mutants with a different effect on ion conduction and lipid scrambling. Similar to previous data from cellular assays (Yu et al., 2015), a construct of mTMEM16F where the SCRD was replaced by the equivalent region of mTMEM16A (mTMEM16F^SCRD^, Figure 4E) exhibited strongly compromised scrambling activity in our in vitro experiments (Figure 6G; Figure 6—figure supplement 2G). In contrast, when investigating the TMEM16F^SCRD^ construct by electrophysiology, we observed robust currents, although with a several-fold reduction in current amplitudes, and an increased potency for Ca^2+^ compared to WT (Figure 6E; Figure 6—figure supplement 2L). Thus, besides the point mutant R478A, also the SCRD chimera appears to exert different effects on lipid and ion permeation, indicating that both processes might be less coupled than previously anticipated (Jiang et al., 2017; Whitlock and Hartzell, 2016; Yu et al., 2015).

## Discussion

By combining data from cryo-electron microscopy, in vitro scrambling assays and electrophysiology, our study has provided insight into the diverse functional behavior of mTMEM16F, a member of the TMEM16 family that facilitates the transport of lipids and ions across cellular membranes. Both processes are controlled by the binding of Ca^2+^ to a site located within the transmembrane domain that is accessible from the cytoplasm and they are mediated by the same structural element located at the periphery of each subunit. Whereas in a cellular environment, the significance of TMEM16F mediated lipid movement is established, the role of ion conduction is currently unclear. Notably, due to the strong outward rectification, ionic currents would be small at resting potential but they could suffice to provide a shunt for the dissipation of charge during the scrambling of anionic lipids, which probably occurs at comparable rates (Watanabe et al., 2018; Yang et al., 2012). Although our results offer detailed insights into potential transport mechanisms, there are still open questions related to the uncertain correspondence of the Ca^2+^-bound conformation of the protein to a defined functional state. We thus envision three alternative scenarios for ion and lipid permeation. In all scenarios, the structures obtained in Ca^2+^-free conditions describe an inactive conformation of the protein, while the Ca^2+^-bound structures either represent an intermediate or final state of activation. Whereas, in light of our cryo-EM structures, all three scenarios appear plausible, the combination with functional data points towards a mechanism where ion conduction and scrambling are mediated by distinct protein conformations.

**Figure 7.**
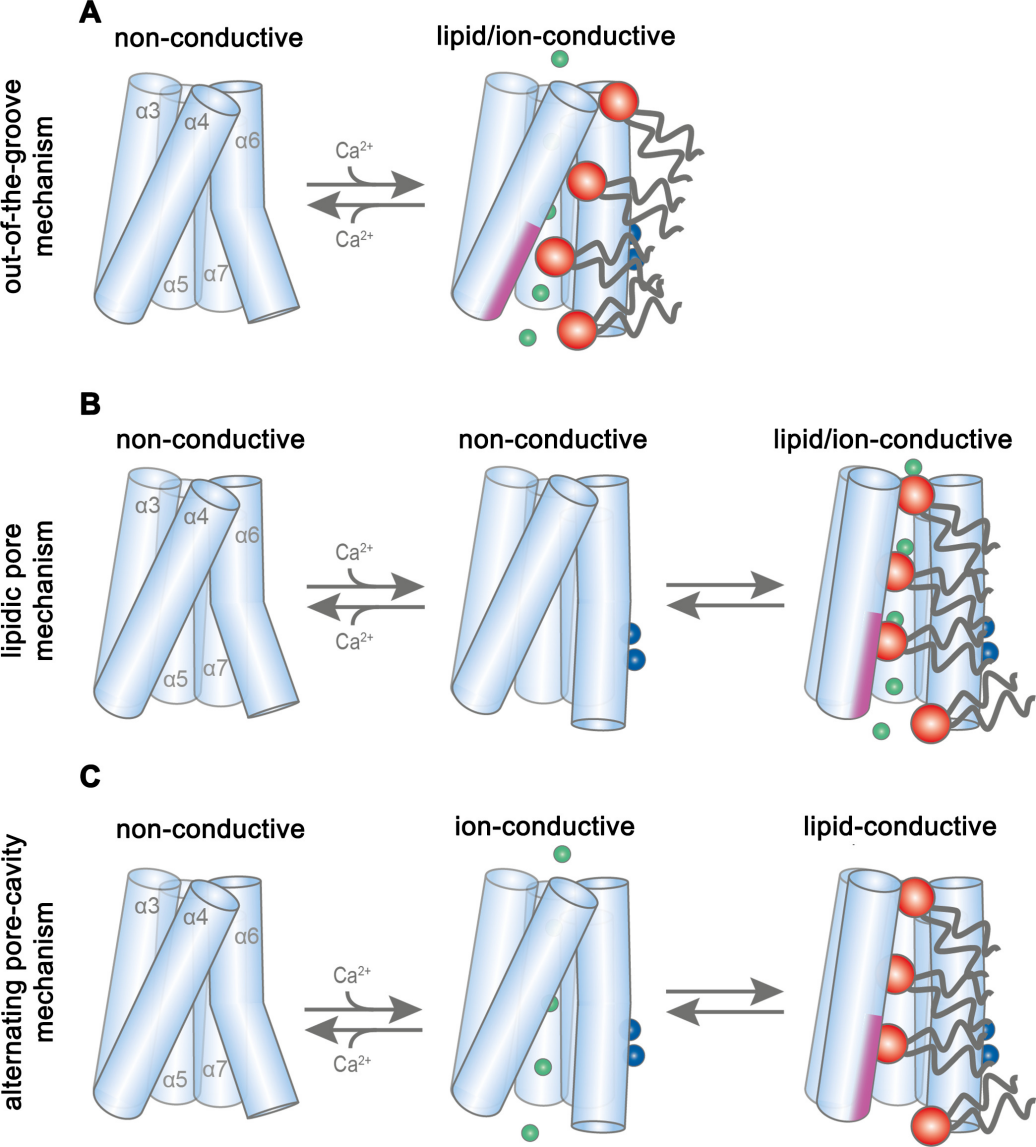
Potential transport mechanisms in mTMEM16F. (**A**) ‘Out-of-the-groove mechanism’: upon calcium-activation the subunit cavity remains closed, ions and lipids permeate separate parts of the protein. (**B**) ‘Lipidic pore mechanism’: upon Ca^2+^ binding, the cavity opens with ions and lipids permeating the same open cavity conformation. (**C**) ‘Alternating pore-cavity mechanism’: After Ca^2+^-binding the open lipid-conductive cavity and ion conductive protein-surrounded pore are in equilibrium. This mechanism appears most likely in light of the functional data on mTMEM16F and the results on a related study on the activation of the lipid scramblase nhTMEM16 (Kalienkova et al., 2019). A-C, Schematic representation of mTMEM16F α3–7 depicted as cylinders, bound calcium ions as blue spheres, conducting chloride ions as green spheres, scrambling lipid head groups as red spheres and their respective acyl chains as grey lines. The scrambling domain (SCRD) is indicated as purple area at the intracellular side of α-helix 4.

In the first scenario (‘out-of-the-groove mechanism’), the here reported Ca^2+^-bound mTMEM16F structures represent a conformation close to the endpoint of the protein activation (Figure 7A). Since, in the Ca^2+^-bound state, the extracellular part of the subunit cavity remains shielded from the membrane, the diffusion of lipids would proceed outside of the cavity for about two-thirds of their transition. In this scenario, scrambling would be facilitated by interactions with the SCRD, which exposes polar residues to the inner leaflet of the bilayer at the gap between α4 and α6. Protein interactions with the lipid headgroups destabilize the membrane and consequently lower the barrier for lipid movement, which does not proceed in tight contact with the protein, as proposed in a recent study (Malvezzi et al., 2018). By contrast, ion conduction takes place in a sufficiently expanded protein-enclosed pore separated from the scrambling path. Upon activation, the observed movement of α6 towards α4 would change the structure in vicinity of the SCRD sufficiently to allow both lipid movement and ion conduction. This scenario would imply that there are two classes of TMEM16 scramblases acting by distinct mechanisms. The first class would allow the passage of lipids through a membrane-accessible furrow and include fungal scramblases (Brunner et al., 2014; Falzone et al., 2019; Kalienkova et al., 2019) and their mammalian orthologues residing in intracellular organelles, such as hTMEM16K (Bushell et al., 2018). A second class, defined by mTMEM16F and its orthologues, which are located in the plasma membrane, are closely related to the ion channels mTMEM16A and B and thus show pronounced ion conduction properties but do not contain a membrane-accessible polar subunit cavity.

In a second scenario, scrambling would proceed on the surface of a subunit cavity that has opened to the membrane (Figure 7B,C). In this case, the Ca^2+^-bound mTMEM16F structures display intermediates, whereby the conformational heterogeneity of α3 and α4 observed in the nanodisc samples indicates a potential rearrangement towards a fully active protein conformation (Figure 2—figure supplements 3I and 4I). Consequently, the comparison to Ca^2+^-free structures reveals the first ligand-dependent activation step. Full activation of the protein to a scrambling-competent state requires a consecutive transition that might be facilitated by lipid interactions with the protein at the SCRD, thereby facilitating the conformational change of α4 (Lee et al., 2018). This transition would lead to the exposure of a membrane-spanning hydrophilic furrow as observed in the scramblase nhTMEM16 (Brunner et al., 2016). This scenario is generally consistent with the activation mechanism for the fungal scramblases nhTMEM16 (Kalienkova et al., 2019) and afMEM16 (Falzone et al., 2019) and the human scramblase TMEM16K (Bushell et al., 2018). The fully active conformation was not observed in our structures, since it is either transient, as proposed for lipid scrambling in rhodopsin (Morra et al., 2018), or disfavored in our lipid nanodiscs, potentially due to an impairing lipid composition or the absence of interacting components (Schreiber et al., 2018; Ye et al., 2018). In such a scenario, we foresee two potential alternatives for the observed ion conduction. Conduction could either proceed in the same conformation as scrambling, in a pore that is partially lined by translocating lipids and thus be directly coupled to lipid movement (‘lipidic pore mechanism’), as suggested in previous investigations (Jiang et al., 2017; Whitlock and Hartzell, 2016) (Figure 7B). Alternatively, ion conduction and scrambling could be mediated by distinct alternating conformations, which are at equilibrium in the Ca^2+^-bound state (Falzone et al., 2018; Kalienkova et al., 2019). Here, scrambling proceeds in an open subunit cavity, as observed for nhTMEM16, afTMEM16 and hTMEM16K, while ion conduction is catalyzed by a protein-enclosed pore that resembles the Ca^2+^-bound mTMEM16F and mTMEM16A structures (‘alternating pore-cavity mechanism’, Figure 7C).

Whereas our structures do not show an opened subunit cavity, the functional data are most consistent with distinct conformations facilitating ion conduction and scrambling, as described in the ‘alternating pore-cavity mechanism’. In this case, mutations could shift the equilibrium between both states and have opposite effects on either function. This is observed for the mutants R478A and TMEM16F^SCRD^, which both mediate Ca^2+^-activated currents with smaller magnitude but increased ligand potency compared to WT, while both show impaired lipid permeation properties (Figure 6E–G). Particularly, this scenario would account for the strong effect of the mutation of Arg 478 on lipid permeation (Figure 6F) (Gyobu et al., 2017), which in our structures is buried in the protein but would become exposed to the membrane in an open cavity. In contrast, the mutation of polar residues on the outside of the subunit cavity, which would contribute to scrambling in the ‘out-of-the-groove mechanism’, have little impact on lipid movement (Figure 6—figure supplement 2J,K). A shift in the equilibrium of the two states might also underlie the decrease in the open probability of the ion conduction pore at negative voltages (Figure 1C, Figure 1—figure supplement 1G). A definitive resolution of the mechanistic ambiguity will require further investigations, for which our study has provided an important foundation.

## Materials and methods

**Key resources table:** 

Reagent type (species) or resource	Designation	Source or reference	Identifier	Additional information
Antibody	Mouse monoclonal Anti-c-Myc	Millipore Sigma	Cat#M4439; Clone#9E10	Dilution – 1:5000
Antibody	Peroxidase Affinipure goat anti-mouse IgG	Jackson Immunoresearch	Cat#115-035-146	Dilution – 1:10000
Chemical compound, drug	FuGENE six transfection reagent	Promega	Cat# E2691	
Chemical compound, drug	Hygromycin B	Gibco, Thermo Fisher Scientific	Cat#10687010	
Chemical compound, drug	Tetracycline hydrochloride	Millipore Sigma	Cat#T7660	
Chemical compound, drug	*n***-**dodecyl**-**β**-**d**-** maltopyranoside, Solgrade	Anatrace	Cat#D310S	
Chemical compound, drug	EX-CELL 293 Serum-Free medium	Millipore Sigma	Cat#14571C	
Chemical compound, drug	Fetal bovine serum	Millipore Sigma	Cat#F7524	
Chemical compound, drug	L-glutamine	Millipore Sigma	Cat#G7513	
Chemical compound, drug	Penicillin-streptomycin	Millipore Sigma	Cat#P0781	
Chemical compound, drug	Valproic acid	Millipore Sigma	Cat#P4543	
Chemical compound, drug	HyClone HyCell TransFx-H medium	GE Healthcare	Cat#SH30939.02	
Chemical compound, drug	Poloxamer 188	Millipore Sigma	Cat#P5556	
Chemical compound, drug	Polyethylenimine MAX 40 K	Polysciences	Cat# 24765–1	
Chemical compound, drug	Dulbecco’s Modified Eagle’s Medium - high glucose	Millipore Sigma	D5671	
Chemical compound, drug	Dulbecco’s Phosphate Buffered Saline	Millipore Sigma	D8537	
Chemical compound, drug	Digitonin Reagent USP	PanReac AppliChem	Cat#A1905	
Chemical compound, drug	Calcium nitrate tetrahydrate	Millipore Sigma	Cat#C4955	
Chemical compound, drug	Sodium chloride	Millipore Sigma	Cat#71380	
Chemical compound, drug	HEPES	Millipore Sigma	Cat#H3375	
Chemical compound, drug	Ethylene glycol-bis(2- aminoethylether)-N,N,N′,N′- tetraacetic acid	Millipore Sigma	Cat#03777	
Chemical compound, drug	cOmplete, EDTA-free Protease Inhibitor Cocktail	Roche	Cat#5056489001	
Chemical compound, drug	Digitonin, High Purity - Calbiochem	EMD Millipore	Cat#300410	
Chemical compound, drug	Biotin	Millipore Sigma	Cat#B4501	
Recombinant protein	PNGase F	Raimund Dutzler laboratory	NA	
Commercial assay or kit	Amicon Ultra-4–100 KDa cutoff	EMD Millipore	Cat#UFC8100	
Commercial assay or kit	0.22 μm Ultrafree-MC Centrifugal Filter	EMD Millipore	Cat#UFC30GV	
Chemical compound, drug	1-palmitoyl-2-oleoyl -glycero-3-phosphocholine	Avanti Polar Lipids, Inc	Cat#850457C	
Chemical compound, drug	1-palmitoyl-2-oleoyl-sn- glycero-3-phospho-(1'-rac- glycerol)	Avanti Polar Lipids, Inc	Cat#840457C	
Chemical compound	Diethyl ether	Millipore Sigma	Cat#296082	
Commercial assay or kit	Bio-Beads SM-2 Adsorbents	Bio-Rad	Cat# 1523920	
Chemical compound, drug	Soybean Polar Lipid Extract	Avanti Polar Lipids, Inc	Cat#541602C	
Chemical compound, drug	Cholesterol	Millipore Sigma	Cat#C8667	
Chemical compound	18:1-06:0 NBD-PE	Avanti Polar Lipids, Inc	Cat#810155C	
Chemical compound, drug	18:1-06:0 NBD-PS	Avanti Polar Lipids, Inc	Cat#810194C	
Chemical compound, drug	14:0 NBD-PE	Avanti Polar Lipids, Inc	Cat#810143 C	
Chemical compound, drug	Potassium chloride	Millipore Sigma	Cat#746436	
Chemical compound, drug	Triton X-100	Millipore Sigma	Cat#T9284	
Chemical compound, drug	Sodium dithionite	Millipore Sigma	Cat#157953	
Chemical compound, drug	N-Methyl-D-glucamine	Millipore Sigma	Cat#66930	
Chemical compound, drug	Sulphuric acid 95–97%	EMD Millipore	Cat# 1.00731.1000	
Commercial assay or kit	Borosilicate glass capilliary with filament	Sutter Instrument	Cat#BF150-86-10HP	
Commercial assay or kit	Pierce Streptavidin Plus UltraLink Resin	Thermo Fisher Scientific	Cat#53117	
Commercial assay or kit	Superose 6 10/300 GL	GE Healthcare	Cat#17-5172-01	
Commercial assay or kit	Flp-In T-Rex Core Kit	ThermoFisher Scientific	Cat#K650001	
Commercial assay or kit	Whatman Nuclepore Track-Etched Membranes diam.19mm, pore size 0.4μm, polycarbonate	Millipore Sigma	Cat#WHA800282	
Commercial assay or kit	Microforge	Narishige	NA	
Commercial assay or kit	Axopatch 200B amplifer	Molecular Devices	NA	
Commercial assay or kit	Digidata 1440	Molecular Devices	NA	
Commercial assay or kit	300 mesh Au 1.2/1.3 cryo-EM grids	Quantifoil	Cat#N1-C14nAu30-01	
Cell line (Human)	Flp-In T-REx 293	ThermoFisher Scientific	Cat#R78007	
Cell line (Human)	HEK-293T	ATCC	Cat#CRL-1573	
Cell line (Human)	HEK293S GnTI^-^	ATCC	Cat#CRL-3022	
Recombinant DNA	Mouse mTMEM16F open reading frame	Dharmacon - Horizon Discovery	GenBank#BC060732	
Recombinant DNA	Mammalian expression vector with C-terminal 3C protease cleavage site, Myc tag and streptavidin binding peptide	Raimund Dutzler laboratory	NA	
Recombinant DNA	Mammalian expression vector with C-terminal 3C protease cleavage site, Venus and Myc tags and streptavidin binding peptide	Raimund Dutzler laboratory	NA	
Recombinant DNA	Synthesized mTMEM16F^SCRD^ cDNA	GenScript	NA	
Recombinant DNA	Membrane scaffold protein (MSP) 2N2	Stephen Sligar laboratory	Addgene:Cat#29520	
Software, algorithm	WEBMAXC calculator	Bers et al., 2010	http://maxchelator.stanford.edu/webmaxc/webmaxcS.htm	
Software, algorithm	Axon Clampfit 10.7	Molecular Devices	NA	
Software, algorithm	Axon Clampex 10.6	Molecular Devices	NA	
Software, algorithm	Focus 1.1.0	Biyani et al. (2017)	https://focus.c-cina.unibas.ch/about.php	
Software, algorithm	MotionCorr2 1.1.0	Zheng et al. (2017)	http://msg.ucsf.edu/em/software/motioncor2.html	
Software, algorithm	CTFFIND 4.1	Rohou and Grigorieff (2015)	http://grigoriefflab.janelia.org/ctf	
Software, algorithm	Relion v 2.1 and 3.0	Kimanius et al., 2016 Zivanov et al., 2018	https://www2.mrc-lmb.cam.ac.uk/relion/	
Software, algorithm	Phenix 1.13	Adams et al. (2010)	http://http://phenix-online.org/	
Software, algorithm	Coot 0.8.9.1	Emsley and Cowtan (2004)	https://www2.mrc-lmb.cam.ac.uk/personal/pemsley/coot/	
Software, algorithm	Pymol 2.0	Schrodinger LLC	https://pymol.org/2/	
Software, algorithm	Chimera 1.12	Pettersen et al. (2004)	https://www.cgl.ucsf.edu/chimera/	

### Cell lines

Flp-In T-REx 293 cell lines stably expressing mTMEM16F were adapted to suspension cultures and were grown at 37°C and 5% CO_2_ in EX-CELL 293 Serum-free medium supplemented with 1% fetal bovine serum, 6 mM L-glutamine and 100 U/ml penicillin–streptomycin. GnTI^-^ cells were grown in HyClone HyCell TransFx-H medium supplemented with 1% fetal bovine serum, 4 mM L-glutamine, 1 g/l poloxamer 188 and 100 U/ml penicillin–streptomycin. Adherent HEK293T cells were grown in DMEM medium supplemented with 10% fetal bovine serum, 2 mM L-glutamine, 1 mM Sodium pyruvate and 100 U/ml penicillin–streptomycin. Mycoplasma test performed on the used cells was negative.

### Construct preparation

The gene encoding mTMEM16F (GenBank: BC060732) was obtained from Dharmacon-Horizon Discovery and stably inserted into a tetracycline-inducible HEK293T cell line using the Flp-In T-Rex System. For that purpose, the mTMEM16F sequence was cloned into two pcDNA5/FRT expression vectors, one with C-terminal Rhinovirus 3C protease recognition site, Myc and Streptavidin-binding peptide (SBP) tags and the other with an extra Venus tag before the Myc and SBP tags. Both cell lines were created in the same manner: briefly, 1 µg of mTMEM16F expression vector and 9 µg pOG44 recombinase were co-transfected after mixing with 30 µg Fugene6 transfection reagent. Hygromycin (50 µg/ml in the first week and 100 µg/ml afterwards) was used as selection marker and resistant foci appeared after 2 weeks. Per cell line, 15 foci were expanded and mTMEM16F expression was assayed after tetracycline induction (2 µg/ml) for 48 hr, extraction with 2% *n***-**dodecyl**-**β**-**d**-**maltopyranoside (DDM) and Western blot analysis using a mouse monoclonal anti-Myc and a Peroxidase Affinipure goat anti-mouse IgG antibodies (Figure 1—figure supplement 1A).

For mutagenesis, the sequence of mTMEM16F was cloned into FX-cloning compatible pcDNA3.1 vectors (Geertsma and Dutzler, 2011) and point mutations were introduced with the non-overlapping primers modified QuikChange method (Zheng et al., 2004). The sequence was not codon optimized except for the removal of a *SapI* restriction site. For experiments requiring protein purification, the sequence was cloned into an expression vector with C-terminal 3C recognition site, Myc and SBP tags. For electrophysiology experiments, an expression vector with either a Venus (in the case of WT and mutants E624Q, E667Q and Y563S) or a GFP tag (in the case of mutants Q559K, G615A, R478A and mTMEM16F^SCRD^) between the 3C site and Myc-SBP was used instead. All constructs were verified by sequencing. The mTMEM16F^SCRD^ construct, with residues 525–559 mutated to their equivalent in mTMEM16A, was synthesized by GenScript. The plasmid used for the expression of the membrane scaffold protein (MSP) 2N2 was obtained from Addgene (plasmid #29520).

### Protein expression and purification

Wild-type mTMEM16F was expressed by tetracycline induction (2 µg/ml) of the stably transfected mTMEM16F-3C-Myc-SBP cell line for 48–70 hr. After induction, the medium was further supplemented with 3.5 mM valproic acid. Mutant mTMEM16F constructs were expressed by transient transfection of GnTI^–^ cells. For cell transfection, DNA was complexed in a 1:2.5 ratio with Polyethylenimine MAX 40 K in non-supplemented DMEM medium for 20 min before addition to the cells. After transfection, the medium was further supplemented with 3.5 mM valproic acid and the cells were collected after 48–60 hr, washed with PBS and stored at −80°C until further use.

Protein purification of wild-type and mutant mTMEM16F was carried out at 4°C and was completed within 14 hr. In all cases, we have purified the protein under Ca^2+^-free conditions and added 1 mM Ca^2+^ when indicated during cryo-EM sample preparation. Cells were resuspended in 2% digitonin (PanReac AppliChem), 150 mM NaCl, 20 mM HEPES, pH 7.5, 5 mM EGTA and protease inhibitors and the membranes were solubilized by gentle mixing for 2 hr. Solubilized proteins were isolated by centrifugation at 85 000 *g* for 30 min and allowed to bind to streptavidin UltraLink resin beads for 2 hr under gentle agitation. The beads were loaded onto a gravity column and washed with 60 column volumes (CV) of SEC buffer containing 0.1% digitonin (EMD Millipore), 150 mM NaCl, 20 mM HEPES, pH 7.5 and 2 mM EGTA. Bound protein was eluted with 3 CV of SEC buffer supplemented with 4 mM biotin. Samples used for cryo-EM were digested with PNGaseF for 2 hr. Protein was concentrated using a 100 kDa cutoff filter, filtered through a 0.22 µm filter and loaded onto a Superose 6 10/300 GL column pre-equilibrated with SEC buffer. Protein-containing peak fractions were pooled, concentrated, filtered and immediately used for either cryo-EM sample preparation or reconstitution into nanodiscs or liposomes. The MSP 2N2 protein was expressed and purified as described (Ritchie et al., 2009). In this case, the N-terminal poly-histidine tag was not cleaved after purification.

Nanodisc reconstitution was performed as described (Gao et al., 2016) with minor modifications. Purified protein was incorporated into nanodiscs composed of a1-palmitoyl-2-oleoyl-glycero-3-phosphocholine (POPC) and 1-palmitoyl-2-oleoyl-sn-glycero-3-phospho-(1'-rac-glycerol) (POPG) mixture at a molar ratio of 3:1. After mixing, the chloroform-dissolved lipids were initially dried under a nitrogen stream, washed with diethyl ether and again dried under nitrogen stream and vacuum desiccation overnight. The lipid mix was solubilized in 30 mM DDM, at a final lipid concentration of 10 mM. A molar ratio of 2:10:2200 of mTMEM16F:MSP 2N2:lipids was used for reconstitution. Purified protein was incubated with the lipid mix on ice for 40 min. Subsequently, 2N2 was added to the mixture and further incubated for 40 min on ice. Detergent was removed overnight after addition of 50 mg of SM-II biobeads per mg of DDM. Biobeads were removed from the clear sample by column filtration and the nanodisc suspension was incubated with streptavidin UltraLink resin for 2 hr. Further purification proceeded as described for the digitonin-solubilized protein, except that no detergent was present in any of the buffers.

### Liposome reconstitution and scrambling experiments

For the characterization of lipid scrambling, purified mTMEM16F was reconstituted into liposomes containing trace amounts (0.5% weight/weight) of fluorescently (nitrobenzoxadiazole, NBD) labeled lipids. The lipid mix used for the majority of scrambling experiments was soybean polar lipids extract with 20% cholesterol (mol/mol) and 0.5% 18:1-06:0 NBD-PE. To investigate whether scrambling proceeds independently of the chemical nature of the headgroup of the labeled lipid or the location of the fluorophore, the tail labeled 18:1-06:0 NBD-PE was substituted by 18:1-06:0 NBD-PS or head-labeled 14:0 NBD-PE while maintaining the other liposome components unchanged. To characterize the protein activity in the lipid mix used for the preparation of nanodisc samples, we reconstituted mTMEM16F into liposomes composed of 3 POPC:1 POPG: 0.5% 18:1-06:0 NBD-PE. Lipids were treated similarly as the ones used for nanodisc reconstitution until solubilization. Liposomes were prepared as described (Geertsma et al., 2008). For liposomes, the lipids were solubilized in 20 mM HEPES pH 7.5, 300 mM KCl and 2 mM EGTA (buffer A) in a final concentration of 20 mg/ml, sonicated, subjected to three freeze-thaw cycles in liquid N_2_ and stored at −80°C. The liposomes were protected from direct light as much as possible. Prior to reconstitution, the lipids were extruded 21 times through a 400 nm pore polycarbonate membrane and diluted to 4 mg/ml in buffer A. Liposomes were destabilized by addition of Triton X-100 aliquots in 0.02% steps until the onset of lipid solubilization monitored by the decrease of the scattering at 540 nm in a spectrophotometer. After addition of another aliquot of 0.1% Triton X-100, the protein was added at a lipid-to-protein ratio of 100:1 (weight/weight) unless stated otherwise to the destabilized liposomes and the mixture was incubated at room temperature (RT) for 15 min under gentle agitation. Subsequently, 20 mg of SM-II biobeads per mg of lipids were added four times to completely remove digitonin and Triton X-100. After 45 min, the sample was moved to 4°C. Proteoliposomes were harvested after 24 hr by filtration to remove the biobeads followed by centrifugation at 150,000 *g* for 30 min. The pelleted proteoliposomes were resuspended in buffer A with solutions containing the appropriate amount of Ca(NO_3_)_2_ to obtain the indicated concentrations of free Ca^2+^ calculated with the online WEBMAXC calculator (Bers et al., 2010). The liposomes were resuspended at either 10 or 20 mg/ml and subjected to three freeze-thaw cycles in liquid N_2_. All remaining steps were carried out at RT. After extrusion through a 400 nm membrane pre-equilibrated in buffer A containing the desired amount of free Ca^2+^, proteoliposomes were diluted to 0.2 mg/ml in 80 mM HEPES pH 7.5, 300 mM KCl, 2 mM EGTA plus Ca^2+^ (buffer B). Scrambling was performed essentially as described for other scramblases of the TMEM16 family (Brunner et al., 2014; Malvezzi et al., 2013). Scrambling was monitored by spectrofluorimeter measurements of the NBD fluorophore with an excitation wavelength of 470 nm and emission wavelength of 530 nm. After 60 s of recording, 30 mM of the membrane-impermeable reducing agent sodium dithionite was added to irreversibly bleach exposed NBD groups. The total recording time was of 400 s unless stated otherwise and the sample was stirred during the entire measurement. The NBD-fluorescence decay was plotted as F/F_max_. To investigate the ligand-dependence of the scrambling rate, we measured the NBD-fluorescence value 120 s after the addition of dithionite at different Ca^2+^ concentrations and quantified scrambling activity as 1-F_Ca2+_/F_0Ca2+_ since we were unable to detect any pronounced scrambling activity for mTMEM16F in the absence of Ca^2+^. The effect of Ca^2+^ on the scrambling rate was studied using symmetric buffer conditions, with the same Ca^2+^ concentration inside and outside the liposomes. To investigate the delay time between calcium addition and scrambling activation we reconstituted mTMEM16F in calcium-free liposomes and recorded the NBD-fluorescence decay upon addition of 100 µM Ca(NO_3_)_2_ to the outside buffer, 120 s after dithionite addition, and monitored fluorescence for extra 420 s. The same batch of liposomes was incubated in buffer B containing 100 µM Ca(NO_3_)_2_ for 10 min prior to measurement as control.

In empty liposomes, addition of dithionite causes a decrease of the fluorescence to about half of its initial value, due to the bleaching of the fraction of fluorescent lipids located in the outer leaflet of the bilayer (Figure 1—figure supplement 1C). In contrast, proteoliposomes containing an active scramblase show a stronger time-dependent decrease of the fluorescence to a plateau, which corresponds to the fraction of fluorescent lipids that are not accessible to dithionite since they either reside in liposomes not containing a reconstituted scramblase or they are located on the inside of a multi-lamellar vesicle (Malvezzi et al., 2018; Ploier and Menon, 2016) (Figure 1A; Figure 1—figure supplement 1C–F). For different reconstitutions, we find similar plateau values for empty liposomes and proteoliposomes in Ca^2+^-free conditions. In contrast, the plateau of fully activated samples is consistent for different constructs using the same batch of destabilized lipids but it varies between different reconstitutions, with fluorescence levels ranging from 18% to 30%. We attribute this difference to the efficiency of reconstitution that is dependent on the batch of destabilized liposomes. Independent of the plateau, all reconstitutions showed a very similar Ca^2+^-dependence. Due to the variable reconstitution efficiency, all the mutants were purified in parallel with wild type (WT) mTMEM16F and reconstituted using the same batch of lipids in equivalent reconstitution conditions. Protein incorporation into liposomes for WT and mutants was verified by Western blot against the Myc tag after solubilization of proteoliposomes with DDM. With the exception of mTMEM16F^SCRD^, all mutants reconstituted with an efficiency similar to WT (Figure 6—figure supplement 2G). mTMEM16F^SCRD^ behaved well during purification but reconstituted with somewhat lower efficiency. For that purpose, it was compared to WT reconstituted at lower lipid to protein ratio of 150:1. For this case proteoliposomes contained similar protein levels (Figure 6—figure supplement 2G).

### Electrophysiology

For electrophysiology, HEK293T cells were transfected with plasmids at a concentration of 8 µg DNA per 100 mm culture dish with 25 µl FuGENE 6 transfection reagent. Transfected cells were identified by Venus or GFP fluorescence and used for patch clamp experiments within 24–72 hr of transfection. WT currents were also recorded from cells stably expressing mTMEM16F without fluorescent fusion protein used for purification. All recordings were performed in the inside-out configuration (Hamill et al., 1981) at RT (20–22°C). Inside-out patches were excised from HEK293T cells expressing the mTMEM16F construct of interest after the formation of a gigaohm seal. Seal resistance was typically 4–8 GΩ or higher. Patch pipettes were pulled from borosilicate glass capillaries (OD 1.5 mm, ID 0.86 mm) and were fire-polished using a microforge. Pipette resistance was typically 3–8 MΩ when filled with pipette solutions. Voltage-clamp recordings were performed using the Axopatch 200B amplifier controlled by the Clampex 10.6 software through Digidata 1440. The data were sampled at 10 kHz and filtered at 1 kHz. Solution exchange was achieved using a double-barreled theta glass pipette mounted on an ultra-high speed piezo-driven stepper (Siskiyou). Liquid junction potential was not corrected. Step-like solution exchange was elicited by analogue voltage signals delivered through Digidata 1440. All recordings were measured in symmetrical NaCl solutions except for selectivity experiments. The background current was recorded in Ca^2+^-free solution and subtracted prior to analysis. Ca^2+^-free intracellular solution contained 150 mM NaCl, 5 mM EGTA, and 10 mM HEPES, pH 7.40. High intracellular Ca^2+^ solution, with a free concentration of 1 mM, contained 150 mM NaCl, 5.99 mM Ca(OH)_2_, 5 mM EGTA, and 10 mM HEPES, pH 7.40. The pH was adjusted using 1 M NMDG-OH solution. Intermediate Ca^2+^ solutions were obtained by mixing high Ca^2+^ and Ca^2+^-free intracellular solutions at the ratio calculated according to the WEBMAXC calculator (Bers et al., 2010), free Ca^2+^ concentrations above 1 mM (up to 10 mM) were adjusted by adding CaCl_2_ from a 1 M stock solution. The Ca^2+^-free intracellular solution was also used as the pipette solution. For permeability experiments using (NMDG)_2_SO_4_ to compensate for the ionic strength, the appropriate NaCl concentration was adjusted by mixing NaCl stock solutions and (NMDG)_2_SO_4_ stock solutions at the required ratios. Stock NaCl solutions were the same as above. Stock (NMDG)_2_SO_4_ solutions contained 100 mM (NMDG)_2_SO_4_, 5.99 mM Ca(OH)_2_, 5 mM EGTA, 10 mM HEPES, pH 7.40, and 100 mM (NMDG)_2_SO_4_, 5 mM EGTA, and 10 mM HEPES, pH 7.40.

Current of mTMEM16F runs down in the excised patch configuration. In order to obtain a more accurate EC_50_ of Ca^2+^-activation, rundown correction was performed using a previously described method (Lim et al., 2016). In short, a reference Ca^2+^ pulse was applied at a regular time interval before and after the test pulse. The magnitude of the test pulse was normalized to the average magnitude of the pre- and post-reference pulses. The normalized concentration–response data were fitted to the Hill equation. To correct for rundown during rectification experiments, the instantaneous and steady-state current at each voltage step was divided by the ratio of the remaining current at the 80 mV pre-pulse and expressed as normalized current (I/I_80mV_). The rectification indices of instantaneous currents were calculated as the ratio between the current measured at 80 mV, 100 ms before the voltage jump, and 6 ms after changing to −80 mV at which the capacitive transients have already decayed. For calculation of the rectification index of steady-state currents, the value at −80 mV was taken once the current response has reached a plateau (typically 150–200 ms after the voltage change).

The significance of differences between the constructs was determined with a one-way ANOVA and a Tukey-Kramer post-hoc test. Values were considered significantly different if p<0.05. By this criterion, shifts in the EC_50_ for mutants E667Q, G615A and TMEM16F^SCRD^ compared to WT were determined as significant whereas the shift in R478A is slightly above the significance threshold. For the characterization of rectification properties of steady-state currents, only the shift of the mutant R478A compared to WT was calculated to be statistically significant. In case of the mutant Q559K, the high variance due to the presence of two populations of data with strong and weak rectification properties rendered the shift in its value as statistically insignificant.

### Cryo-electron microscopy sample preparation and imaging

2.5 μl of freshly purified protein at a concentration of 3.3 mg ml^–1^, when solubilized in digitonin, and 1 mg ml^–1^, when reconstituted in nanodiscs, were applied on holey-carbon cryo-EM grids (Quantifoil Au R1.2/1.3, 200, 300 and 400 mesh), which were prior glow-discharged at 5 mA for 30 s. For the datasets obtained for the Ca^2+^-bound structures, samples were supplemented with 1 mM CaCl_2_ before freezing. Grids were blotted for 2–5 s in a Vitrobot (Mark IV, Thermo Fisher) at 10–15°C temperature and 100% humidity, subsequently plunge-frozen in liquid ethane and stored in liquid nitrogen until further use. Cryo-EM data were collected on a 200 keV Talos Arctica microscope (Thermo Fisher) using a post-column energy filter (Gatan) in zero-loss mode, a 20 eV slit, a 100 μm objective aperture, in an automated fashion using EPU software (Thermo Fisher) on a K2 summit detector (Gatan) in counting mode. Cryo-EM images were acquired at a pixel size of 1.012 Å (calibrated magnification of 49,407x), a defocus range from –0.5 to –2 μm, an exposure time of 9 s with a sub-frame exposure time of 150 ms (60 frames), and a total electron exposure on the specimen of about 52 electrons per Å^2^. Best regions on the grid were manually screened and selected with the help of an in-house script to calculate the ice thickness (manuscript in preparation). Data quality was monitored on the fly using the software FOCUS (Biyani et al., 2017).

### Image processing

For the detergent dataset collected in presence of Ca^2+^, the 5633 dose-fractionated cryo-EM images recorded (final pixel size 1.012 Å) were subjected to motion-correction and dose-weighting of frames by MotionCor2 (Zheng et al., 2017). The CTF parameters were estimated on the movie frames by ctffind4.1 (Rohou and Grigorieff, 2015). Bad images showing contamination, a defocus above –0.5 or below –2 μm, or a bad CTF estimation, were discarded. The resulting 4225 images were used for further analysis with the software package RELION2.1 (Kimanius et al., 2016). Particles were initially picked automatically from a subset of the dataset using 2D-class averages from the previously obtained mTMEM16A cryo-EM map (Paulino et al., 2017a) as reference. The resulting particles where used to create a better reference for autopicking, which was then repeated on the whole dataset. The final round of autopicking yielded 1,348,247 particles, which were extracted with a box size of 220 pixels, and initial classification steps were performed with two-fold binned data. False positives were removed in the first round of 2D classification. Remaining particles were subjected to several rounds of 2D classification, resulting in 680,465 particles that were further sorted in several rounds of 3D classification. The mTMEM16A cryo-EM map, low-pass filtered to 50 Å, was used as a reference for the first round of 3D classification and the best output class was used in subsequent jobs in an iterative way. The best 3D classes, comprising 219,302 particles, were subjected to auto-refinement, yielding a map with a resolution of 3.8 Å. In the last refinement iteration, a mask excluding the micelle was used and the refinement was continued until convergence, which improved the resolution up to 3.5 Å. Using a mask generated from the final PDB model, we obtained a map at 3.4 Å. Finally, the newly available algorithms for CTF refinement and Bayesian polishing implemented in Relion3.0 (Zivanov et al., 2018)., where applied to further improve the resolution to 3.18 Å with a map sharpened at –94 Å^2^. During final 3D classification and auto-refinement jobs, a C2-symmetry was imposed. Local resolution was estimated by RELION. All resolutions were estimated using the 0.143 cut-off criterion (Rosenthal and Henderson, 2003) with gold-standard Fourier shell correlation (FSC) between two independently refined half maps (Scheres and Chen, 2012). During post-processing, the approach of high-resolution noise substitution was used to correct for convolution effects of real-space masking on the FSC curve (Chen et al., 2013). The directional resolution anisotropy of density maps was quantitatively evaluated using 3DFSC (Biyani et al., 2017).

For the other datasets, a similar workflow for image processing was applied. In the case of the detergent dataset collected in absence of Ca^2+^, a total of 1,314,676 particles were extracted after autopicking from 4621 images. Several rounds of 2D and 3D classification resulted in a final number of 194,284 particles, which after refinement yielded a 4.1 Å map. Providing a mask in the last iteration of the refinement, the resolution was improved to 3.8 Å. After post-processing the resolution improved to 3.6 Å. For the dataset in nanodiscs in the presence of Ca^2+^, 1,019,012 particles were picked from 4480 images and extracted with a box size of 256 pixels. The final set of 186,487 particles was refined, yielding a map with a resolution of 3.9 Å, which after post-processing was improved to 3.5 Å. Finally, the dataset in nanodisc in absence of Ca^2+^ resulted in 1,593,115 autopicked particles from 6465 images, which were reduced to 280,891 particles after several rounds of 2D and 3D classification. Refinement was performed providing a mask in the last iteration, reaching a resolution of 3.3 Å after post-processing.

The analysis of structural heterogeneity within each dataset was conducted by running an additional 3D classification step with finer angular sampling on the final particle set. While some structural flexibility was revealed for α3 and α4 in the dataset in presence of Ca^2+^ (Figure 2—figure supplements 1I and 3I), no alternative conformations could be identified in the dataset in absence of Ca^2+^ (Figure 2—figure supplements 2I and 4I), as reflected in the better resolved density of the helices (Figure 2—figure supplement 5).

Finally, as revealed by the angular distribution and the 3D FSC plots obtained for the data in nanodiscs, the samples suffered from favorite orientation of the particles in vitreous ice, thereby compromising to some extent the detailed features of the maps (Figure 2—figure supplements 3–5). The problem is less pronounced in the nanodisc data obtained in presence of Ca^2+^ than in the dataset of the Ca^2+^-free sample. Nevertheless, the data in nanodiscs are of sufficient quality to allow for a comparison of corresponding states of mTMEM16F in detergent and a lipid environment.

### Model building refinement and validation

The model of the Ca^2+^-bound state of mTMEM16F in digitonin was built in COOT (Emsley and Cowtan, 2004), using the structure of mTMEM16A (PDBID 5YOB) as template. The density was of sufficiently high resolution to unambiguously place the model consisting of residues 43–81, 87–149, 202–223, 228–427, 445–488, 503–587, 590–640, 645–791, 795–875. A DPPC (1,2-didecanoyl-sn-glycero-3-phosphocholine-ligand ID P1O) molecule was fitted into unassigned electron density close to the dimer interface of mTMEM16F, although the exact lipid species could not be determined. Coordinates were manually edited in COOT (Emsley and Cowtan, 2004) and the model was improved by real-space refinement in Phenix (Adams et al., 2010), whereby secondary structure elements and the symmetry between both subunits of the dimeric protein were constrained. The models of the Ca^2+^-bound (PDBID 6QP6) and Ca^2+^-free (PDBID 6QPB) structure in digitonin and the Ca^2+^-bound structure obtained in nanodiscs (PDBID 6QPC) of mTMEM16F were built using the refined Ca^2+^-bound structure as starting model. Major conformational changes were restricted to the second half of α6 and to some extent to α3 and α4. These regions were rebuilt manually in COOT (Emsley and Cowtan, 2004) and subsequently refined in Phenix (Adams et al., 2010), as described above. Due to the strong anisotropy of the dataset of mTMEM16F obtained in nanodiscs in absence of calcium, only the transmembrane domain, as a poly-alanine model, was interpreted (PDBID 6QPI). For validation of the refinement, Fourier shell correlations (FSC) between the refined model and the final map were determined (FSC_SUM_, Figure 2—figure supplements 1E, 2E, 3E and 4E). To monitor the effects of potential over-fitting, random shifts (up to 0.3 Å) were introduced into the coordinates of the final model, followed by refinement in Phenix (Adams et al., 2010) against the first unfiltered half-map. The FSC between this shaken-refined model and the first half-map used during validation refinement is termed FSC_work_, the FSC against the second half-map, which was not used at any point during refinement, FSC_free_. The marginal gap between the curves describing FSC_work_ and FSC_free_ indicates no over-fitting of the model. The higher resolution of α-helix three obtained in the cryo-EM map of mTMEM16F in digitonin in absence of Ca^2+^ allowed the unambiguous assignment of its residues. Based on this structure and the conservation between both paralogs, we remodeled α3 of mTMEM16A (Paulino et al., 2017a), which in its original data was poorly resolved. In the new model of mTMEM16A, the register of α3 has shifted by three residues and Arg 515 now superimposes with Arg 478 in mTMEM16F, both pointing into the pore lumen (Figure 4B; Figure 4—figure supplement 1A). The corrected model of mTMEM16A was used for structural comparisons throughout the manuscript. The pore diameter was calculated in HOLE (Smart et al., 1996). Pictures were generated using pymol (The PyMOL Molecular Graphics System, Version 2.0 Schrödinger, LLC), chimera (Pettersen et al., 2004) and chimeraX (Goddard et al., 2018).

### Electrostatic calculations

The electrostatic potential was calculated as described (Paulino et al., 2017a). The linearized Poisson–Boltzmann equation was solved in CHARMM (Brooks et al., 1983; Im et al., 1998) on a 200 Å ×140 Å × 190 Å grid (1 Å grid spacing) followed by focusing on a 135 Å x 100 Å x 125 Å grid (0.5 Å grid spacing). Partial protein charges were derived from the CHARMM36 all-hydrogen atom force field. Hydrogen positions were generated in CHARMM. The protein was assigned a dielectric constant (*ϵ*) of 2. Its transmembrane region was embedded in a 30-Å-thick slab (*ϵ* = 2) representing the hydrophobic core of the membrane and two adjacent 15-Å-thick regions (*ϵ* = 30) representing the headgroups. This region contained a cylindrical hole around the water-filled intracellular vestibule of one subunit and was surrounded by an aqueous environment (*ϵ* = 80) containing 150 mM of monovalent mobile ions.

### Statistics and reproducibility

Electrophysiology data were repeated multiple times from different transfections with very similar results. Conclusions of experiments were not changed upon inclusion of further data. In all cases, leaky patches were discarded. Statistical difference between the constructs were determined with one-way ANOVA and Tukey-Kramer post-hoc test.

## Data Availability

The three-dimensional cryo-EM density maps of calcium-bound mTMEM16F in detergent and nanodiscs have been deposited in the Electron Microscopy Data Bank under accession numbers EMD-4611 and EMD-4613, respectively. The maps of calcium-free samples in detergent and nanodiscs were deposited under accession numbers EMD-4612 and EMD-4614, respectively. The deposition includes the cryo-EM maps, both half-maps, and the mask used for final FSC calculation. Coordinates of all models have been deposited in the Protein Data Bank under accession numbers 6QP6 (Ca^2+^-bound, detergent), 6QPC (Ca^2+^-bound, nanodisc), 6QPB (Ca^2+^-free, detergent) and 6QPI (Ca^2+^-free, nanodisc). The following datasets were generated: AlvadiaCLimNKClericoMosina VOostergetelGTDutzlerRPaulinoC2019Cryo-EM structure of calcium-bound mTMEM16F lipid scramblase in digitoninProtein Databank6QP6 AlvadiaCLimNKClericoMosina VOostergetelGTDutzlerRPaulinoC2019Cryo-EM structure of calcium-free mTMEM16F lipid scramblase in digitoninProtein Databank6QPB AlvadiaCLimNKClericoMosina VOostergetelGTDutzlerRPaulinoC2019Cryo-EM structure of calcium-bound mTMEM16F lipid scramblase in nanodiscProtein Databank6QPC AlvadiaCLimNK2019Cryo-EM structure of calcium-free mTMEM16F lipid scramblase in nanodiscProtein Databank6QPI AlvadiaCLimNKClericoMosina VOostergetelGTDutzlerRPaulinoC2019Cryo-EM structure of calcium-bound mTMEM16F lipid scramblase in digitoninElectron Microscopy Data BankEMD-4611 AlvadiaCLimNKClericoMosina VOostergetelGTDutzlerR2019Cryo-EM structure of calcium-free mTMEM16F lipid scramblase in digitoninElectron Microscopy Data BankEMD-4612 AlvadiaCLimNKClericoMosina VOostergetelGTDutzlerRPaulinoC2019Cryo-EM structure of calcium-bound mTMEM16F lipid scramblase in nanodiscElectron Microscopy Data BankEMD-4613 AlvadiaCLimNKClericoMosina VOostergetelGTDutzlerRPaulinoC2019Cryo-EM structure of calcium-free mTMEM16F lipid scramblase in nanodiscElectron Microscopy Data BankEMD-4614

## Additional files

Transparent reporting form
