# Supplementary material for: Cryo-EM structures and functional characterization of the murine lipid scramblase TMEM16F
Source: eLife. 2019 Feb 20;8:e44365. doi: 10.7554/eLife.44365 (PMC6414204; doi:10.7554/eLife.44365)
Supplement: Transparent reporting form [file elife-44365-transrepform.pdf]

eLIFE
1st Floor
24 Hills Road
Cambridge CB2 1JP, UK
P 01223 855340
W elifesciences.org
T @elife

eLife's transparent reporting form

We encourage authors to provide detailed information *within their submission* to facilitate the interpretation and replication of experiments. Authors can upload supporting documentation to indicate the use of appropriate reporting guidelines for health-related research (see EQUATOR Network), life science research (see the BioSharing Information Resource), or the ARRIVE guidelines for reporting work involving animal research. Where applicable, authors should refer to any relevant reporting standards documents in this form.

If you have any questions, please consult our Journal Policies and/or contact us: editorial@elifesciences.org.

Sample-size estimation

- You should state whether an appropriate sample size was computed when the study was being designed
- You should state the statistical method of sample size computation and any required assumptions
- If no explicit power analysis was used, you should describe how you decided what sample (replicate) size (number) to use

Please outline where this information can be found within the submission (e.g., sections or figure legends), or explain why this information doesn't apply to your submission:

- The sample size for cryo-EM analysis was chosen to obtain the best possible resolution for each dataset. The detailed procedure is outlined in materials and methods section on cryo-EM data collection and image processing
- No sample size was computed when the study was being designed
- No statistical method of sample size computation was used
- For electrophysiology, data were collected until the averaged data have converged (when further addition of data does not change the averaged data). Measurements were based on 3 – 12 cells as described in the figure legends. The corresponding errors were calculated as s.e.m. and are shown in Figure 1D, Figure 1-figure supplement 1, Figures 6B-E and Figure 6-figure supplement 2B.
- For phospholipid scrambling experiments, each construct was independently reconstituted at least two times. Per reconstitution event, three technical replicates were measured. Errors were calculated as s.e.m. and are shown in all figures containing scrambling experiments data.

Replicates

- You should report how often each experiment was performed
- You should include a definition of biological versus technical replication
- The data obtained should be provided and sufficient information should be provided to indicate the number of independent biological and/or technical replicates
- If you encountered any outliers, you should describe how these were handled
- Criteria for exclusion/inclusion of data should be clearly stated

- High-throughput sequence data should be uploaded before submission, with a private link for reviewers provided (these are available from both GEO and ArrayExpress)

Please outline where this information can be found within the submission (e.g., sections or figure legends), or explain why this information doesn't apply to your submission:

- Number of performed experiments is included in the figure legend
- For electrophysiology experiments, number of replicates refers to independent biological replicates. Each experiment was repeated on separate independent transfections.
- For lipid scrambling data, number of biological replicates refers to the number of independent protein reconstitutions. Per reconstitution, three technical replicates were performed. Each experiment was repeated on separate independent protein purification and reconstitution events.
- The data will be provided in accord to eLife's regulations and sufficient information on the number of independent biological replicates have been provided in the figure legends.
- No obvious outliers regarding the particular behavior of independent biological replicates have been encountered and only outliers originating for technical reasons were excluded.
- No high-throughput sequencing data were involved in this manuscript

Statistical reporting

- Statistical analysis methods should be described and justified
- Raw data should be presented in figures whenever informative to do so (typically when N per group is less than 10)
- For each experiment, you should identify the statistical tests used, exact values of N, definitions of center, methods of multiple test correction, and dispersion and precision measures (e.g., mean, median, SD, SEM, confidence intervals; and, for the major substantive results, a measure of effect size (e.g., Pearson's r, Cohen's d)
- Report exact p-values wherever possible alongside the summary statistics and 95% confidence intervals. These should be reported for all key questions and not only when the p-value is less than 0.05.

Please outline where this information can be found within the submission (e.g., sections or figure legends), or explain why this information doesn't apply to your submission:

- For cryo EM maps, the resolution was estimated the gold-standard Fourier shell correlation between two independently refined half-maps. For details, see materials and methods.
- Details on analysis methods are provided in this manuscript. Electrophysiology and lipid scrambling data errors are expressed as s.e.m.. The number of replicates is indicated in the figure legend. Averaged recordings are shown in Figure 1B, 1D, Figure 1-figure supplement 1C-F and I, Figure 6, Figure 6-figure supplement 1A,B, Figure 6-figure supplement 2B,E,F.
- Whenever informative, raw data have been presented in the figures
- For electrophysiology data, statistical difference between the constructs was determined with one-way ANOVA and Tukey-Kramer post-hoc test. Values were considered significantly different if $P < 0.05$.
- No statistical tests were used for lipid scrambling data.

(For large datasets, or papers with a very large number of statistical tests, you may upload a single table file with tests, Ns, etc., with reference to sections in the manuscript.)

Group allocation

- Indicate how samples were allocated into experimental groups (in the case of clinical studies, please specify allocation to treatment method); if randomization was used, please also state if restricted randomization was applied
- Indicate if masking was used during group allocation, data collection and/or data analysis

Please outline where this information can be found within the submission (e.g., sections or figure legends), or explain why this information doesn't apply to your submission:

- Individual reconstitution experiments were treated as separate groups, in which WT protein was compared to mutants. For details, see materials and methods.

Additional data files ("source data")

- We encourage you to upload relevant additional data files, such as numerical data that are represented as a graph in a figure, or as a summary table

- Where provided, these should be in the most useful format, and they can be uploaded as “Source data” files linked to a main figure or table
- Include model definition files including the full list of parameters used
- Include code used for data analysis (e.g., R, MatLab)
- Avoid stating that data files are “available upon request”

Please indicate the figures or tables for which source data files have been provided:

- No numerical data have yet been provided but can be made available upon publication
